# Analyzing land suitability for key cereal crops in Mansa Watershed, Southwest Ethiopia

**DOI:** 10.1371/journal.pone.0328533

**Published:** 2025-08-22

**Authors:** Wude Taye, Wakshum Shiferaw, Genaye Tsegaye

**Affiliations:** Arba Minch University, College of Agricultural Sciences, Natural Resource Management, Arba Minch, Ethiopia; University of Palermo, ITALY

## Abstract

This study is vital for helping farmers to produce appropriate crops based on physical land suitability and for assisting land use planners in decision-making. Large-scale crop production is essential to supply raw materials for industries, focusing on areas with high production potential to boost yields and meet growing demand. However, physical land suitability analysis for major cereal crops is lacking in Mansa Watershed of Southwest Ethiopia. Therefore, this research aimed to assess the physical land suitability for key crops such as wheat, maize, and teff. Utilizing the FAO land evaluation framework, the study employed various data sets, including Sentinel-2A satellite images, soil data, climate information, and elevation models, to determine suitability factors. The Analytical Hierarchy Process (AHP) was used for pairwise comparison of parameters, while Geographic Information System (GIS) software’s weighted overlay tool was applied to evaluate suitability for the specified crops. A vector overlay was utilized for land allocation for each crop. The analysis considered ten criteria: soil pH, depth, texture, drainage, organic matter, slope, altitude, rainfall, temperature, and land use change. Results indicated that approximately 29.6%, 61%, and 50% of the study area were moderately suitable for maize, teff, and wheat production, respectively. Additionally, 52.8%, 38.8%, and 13.9% of the area were marginally suitable for these crops, while 17.6% and 36% of the area were unsuitable for maize and wheat, respectively. Overall, 44% of the land was moderately suitable, and 10% was marginally suitable for the selected crops. Notably, there were no areas classified as highly suitable; most lands were identified as moderately or marginally suitable. Moving forward, sustainable land management practices are necessary to enhance land suitability and improve soil health. Further analyses should also consider irrigation facilities, market access, and processing industries to provide more options for stakeholders and policymakers.

## Introduction

Evaluation of land suitability is crucial for improving food security, both globally and specifically in Ethiopia. Land suitability refers to the fitness of a specific type of land for a particular use. As one of the most important natural resources, land plays a vital role in supporting livelihoods and meeting the increasing demands for food, fiber, feed, and fuel [1]. This is especially true for countries whose economies are primarily based on agriculture. Therefore, land suitability assessment is essential for sustainable land use planning and management [2]. Land suitability assessment can be a key tool for identifying resource potentials and environmental constraints that can provide alternatives for better sustainable land use planning [3]. The physical suitability of land focuses on factors such as climate, land morphology, and soil quality [4]. FAO [5] recommends assessing land suitability for crop production based on climatic, topographical, and soil characteristics. Analyzing land suitability is a valuable tool for planning and managing land resources [6].

Ethiopia faces significant challenges in food security with a growing population and heavy reliance on natural resources [7]. Ethiopia has a rapidly growing population of over 100 million [8], making it one of the fastest-growing populations in Africa. This puts immense pressure on land resources resulted in land degradation and loss of arable land. Therefore, ensuring suitable land for agricultural production is crucial to mitigate the risk of food insecurity and sustain the growing population [9]. In this regard, land suitability assessment is the base for sustainable land resources planning and management [10].

Ethiopia cultivates a variety of crops due to diverse agroecologies, favorable climate conditions, and fertile soils [11]. Cereals like teff, wheat, maize, sorghum, and barley are major staple crops and contribute to food security [12]. However, factors such as inappropriate land use, overgrazing, deforestation, and climate change have contributed to environmental degradation and reduced agricultural outputs. The study area, Mansa watershed, allows for the cultivation of various grains, but poor land use and soil nutrient depletion have led to insufficient crop yields [13].

Adopting land suitability evaluation is essential for sustainable land resource management for instance, through reducing soil erosion to boost agricultural production [14]. Land suitability analysis combines multiple factors to make informed decisions [15]. Analytic hierarchical process (AHP) is one of the most widely used MCDM techniques. This technique is frequently used in conjunction with geographic information systems (GIS) to evaluate ecological capabilities in land suitability and natural resource management, as well as to establish the relative weight of decision criteria [16]. Remote sensing (RS) and geographic information systems (GIS) are examples of machine learning tools that can be effectively combined to create intelligent systems for land use planning [17]. Results from machine learning (ML) techniques are more accurate because they create a new model using statistical analysis and computational algorithms [18]. The operation and representation of geospatial data in suitability analysis has been done in recent years using geographic information systems [19]. These technologies can be used efficiently in systems involving information, coordination, control, and communication. A multitude of outputs, such as maps of land use and cover, obstacles, slope, road mobility, and line-of-sight plots, can be produced using satellite remote sensing data [20].

We identified potentially hostile environments and selected better locations for border control and military strategy using GIS, machine learning, and RS-based techniques [21]. These methods can also yield more comprehensive data and information, which can be used as a foundation for choosing a site. One well-known application using geospatial data is site selection through the use of GIS, RS, analytical hierarchy process, and machine learning [22]. The use of machine learning, the analytical hierarchy method, and GIS are frequently used tools that let users choose Decision-making is aided through the integration of multicriteria evaluation techniques with geographic information systems, which foster the management and organization of vast volumes of geographic data [23]. While multicriteria techniques evaluate alternatives based on the subjective values and priorities of the decision-maker, GIS performs deterministic overlay and buffer operations in site selection problems [24]. Decision-makers can select the most suitable location with the aid of multicriteria decision systems and GIS [25]. GIS and MCE together provide a potent tool for selecting key suitable crop sites and generating excellent analytical outputs.

Furthermore, a computer program used Genetic Algorithm for Rule Set Production (GARP) uses genetic algorithms to build ecological niche models for various species such as cereal crops. The environmental parameters such as heat, precipitation, and elevation, etc., are the species should be able to sustain population levels are described by the generated models. Local species observations and associated environmental parameters are used as input, describing possible boundaries of the species’ capacity for survival. Geographic information systems frequently store such types of environmental parameters. A random collection of mathematical formulas, also known as limiting environmental conditions, makes up a GARP model. Every rule is used as randomly assembled to produce a limitless amount of models that could potentially describe the possibility of the species [26].

The integration of various software including ArcMap, ERDAS imagine, and IDRISI using the Sentinel-2A satellite images. Multi-Criteria Decision Making (MCDM) with Analytical Hierarchy Process (AHP) matrix are calculated which provides decision-makers with relevant maps and location databases to consider arable land for better agricultural production. However, MCDM and AHP are not new algorithms but methods. It has been used in many spatial modeling studies. A branch of operations research known as multiple-criteria decision-making (MCDM) or multiple-criteria decision analysis (MCDA) formally assesses several competing criteria when making decisions. According to Gharye et al. [27], it is also referred to as multi-objective decision analysis, multiple attribute utility theory, multiple attribute value theory, and multiple attribute preference theory. Additionally, the Analytic Hierarchy Process (AHP), a popular MCDM technique that facilitates decision-making, enables decision-makers to rank options based on a set of criteria. An analytical method for classifying and assessing complex decisions is the Analytic Hierarchy Process (AHP). Saaty developed AHP in the 1970s to help decision-makers evaluate and prioritize options based on a set of standards [27]. This study aimed to conduct a physical land suitability analysis using GIS, RS, and integrated multi-criteria to identify suitable areas for teff, wheat, and maize cultivation in Mansa watershed. Factors such as soil, climate, land use/ land cover, and topography were considered. The study seeks to identify the physical factors that determine the suitability of these crops, evaluate the land suitability, and allocate suitable land for each crop within the watershed. The findings of this study will provide valuable insights for decision-makers in sustainable land use planning and agricultural production.

## Materials and methods

### Description of the study area

This study was conducted in Mansa watershed, situated in Dawuro zone of Southwest Ethiopia National Regional State. Geographically, the study area lies between 6°42′ 30”N – 7°4′30”N and 36°53′0”E–37°15′0”E at an altitude ranging from 607 m to 2763 m and having total area of 1024.23 km^2^ [28].

**Fig 1:**
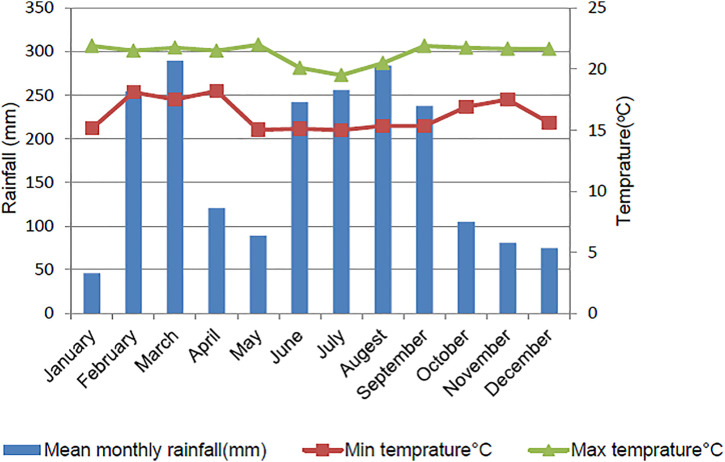
Rainfall and temperature distribution of the study area.

**Climate:** The mean annual temperature varied from 15.02°C to 21.31°C and the average annual rainfall varied between 1529–1663 mm (Fig 1). The study area is characterized by a bimodal rainfall pattern. The short rainy season was between February and March and the long rainy season was between June and September [28].

**Topography and soil:** The Mansa watershed is characterized by diverse topographical features, including valleys, plains, hills, and mountains. The study area comprises six soil types, namely dystric nitisols, dystric fluvisols, dystric gleysols, orthic acrisols, leptosols, and eutric cambisols. Among these, dystric nitisol is the most dominant followed by cambisol and orthic-acrisol [19]. The understanding of soil information is crucial for land use planning.

Demographic and crop production: According to annual statistics from the BoFED [29], the watershed has a population of approximately 73,158, with 37,409 males and 35,749 females [28], with a significant reliance on subsistence agriculture and livestock farming. Dominant crops include maize, teff, and wheat, alongside other produce like barley, enset, and various fruits.

### Research methods

#### Research design and approach.

This research employed a quantitative design, utilizing tables and graphs to represent data from factors such as DEM, rainfall, slope, soil, and temperature, enabling the assessment of suitable land for selected cereal crops.

#### Data source and software used.

This study utilized both spatial and non-spatial data from various organizations (Table 1).

**Table 1:** Summary of data sources and their purpose.

Data set	Format of data	Scale/ Resolution	Source of data	Functions
Digital Elevation Model (DEM)	Tiff	30x30m	https://earthexplorer.usgs	To generate elevation and slope map of the study area
Climate data	MS Excel		Ethiopia National Meteorology Agency (NMA)	To interpolate temperature and rainfall data
Sentinel-2A image of 2021	Tiff	10m*10m	https://earthexplorer.usgs.gov	To develop a land use land cover map of the study area
Soil data	Shape File	at a scale of 1:250,000	Agricultural Transformation Agency (ATA)	To develop soil OM, pH, Depth, Drainage and Texture map of the study area

Various software types, including ArcMap 10.3, ERDAS IMAGINE 2015, IDRISI 17.0, and Google Earth, were employed for a range of activities in the creation of land suitability maps (Table 2).

**Table 2:** Software types and their purposes.

Software used	Purpose
ERDAS IMAGIN 2015	Image processing, data analysis and LULC classification
ArcGIS 10.3	Data processing, calculating, classifying, overlay analysis and map preparation
IDRISI Selva 17.0	For weight derivation of factors
Google earth	Used for visual interpretation tool for accuracy assessment for LULC classification

#### Parameters used for land suitability analysis of cereal crops.

a. **Crop requirement and criteria rating for land suitability**

This study utilized the FAO [5] framework for land suitability analysis, evaluating environmental criteria for maize, teff, and wheat based on climate, topography, and soil characteristics. The criteria were developed from FAO guidelines and existing literature on land suitability for small-scale rain-fed agriculture [5,30–37], summarized in Table 3.

**Table 3:** Environmental requirements for maize, teff, and wheat under rain-fed agriculture.

Crops	Factor	Unit	Range of Suitability			
			S1	S2	S3	N
Teff	Rainfall	Mm	450–550	300–450/ 550–800	800–1200	<200 >1200
	Temperature	^o^c	15–21	14–15/21–22	12–14/ 22–23	<12/>25
	Elevation	M	1600–2200	1000–1600/ 2200–2400	2400–2800	<1000 >2800
	Slope	%	0–7	7–15	15–25	>25
	Soil PH	–	5.5–7.5	5.2–5.5 and 7.5–7.8	5.0–5.2 and 7.8–8.0	<4.5 >8.5
	Soil OM	%	>3	2–3	1–2	<1
	Soil depth	Cm	>50	30–50	20–30	<10
	Soil texture	Class	C,Si,SiC,	SiC,	siL.Cl,SL	L,SCL,
	Soil drainage	Class	W, MM	I	SE, E	P, VP
	LULU	Type	CL	Grassland	BShL	WL,BL
Wheat	Rainfall	Mm	450–650	350–450, 650–850	300–350, 850–1000	<300 >1000
	Temperature	^o^c	14.9–18.4	14.4–14.9 /18.4–19.4	13.4–14.4, 19.4–20.8	<13 >20.8
	Elevation	M	1600–2200	1000–1600/ 2200–2400	2400–2800	<1000 >2800
	Slope	%	0–13	13–25	25–40	>40
	Soil pH	–	6.0–8.0	5.2–6.0 and 8.0–8.3	5.0–5.2 and 8.3–8.5	<5 and >8.5
	Soil OM	%	>3	2–3	1–2	<1
	Soil depth	Cm	>100	75–100	50–75	<50
	Soil texture	Class	C,Si,SiC,SiL,SC	L,CL.SicL	SCL	LS,S
	Soil drainage	Class	W,SE	MW	I	VP
	LULU	Type	CL	Grassland	BShL	WL,BL
Maize	Rainfall	Mm	500–750	450–500/ 750–1200	300–400 1200–1600	<300 >1600
	Temperature	^o^c	15–21	14–15/21–22	12–14/ 22-23	<11/>25
	Elevation	M	1600–2200	1000–1600/ 2200–2400	2400–2800	<1000 >2800
	Slope	%	0–7	7–15	15–25	>25
	Soil pH	–	5.5–7.5	5.2–5.5 and 7.5–7.8	5.0–5.2 and 7.8–8.0	<4.5 >8.5
	Soil OM	%	>3	2–3	1–2	<1
	Soil depth	Cm	>50	30–50	20–30	<10
	Soil texture	Class	SI,SiC,C	Sic	SiL,CL,SC	L,SCL,S,SL
	Soil drainage	Class	W	Mw	VP	E,SE
	LULU	Type	CL	Grassland	BShL	WL,BL

S1 = Highly suitable, S2 = Moderately suitable, S3 = Marginally suitable, N = Not suitable, P = poorly drained, VP = very poorly drained, SP = somewhat poorly drained, MW = moderately well drained = well drained, SE = somewhat excessively drained = excessively drained, I = imperfectly, C = Clay, CL = Clay loam; SCL = Silt-clay-loam, SiL = Silty-loam, L = Loam, SL = Sandy-loam, CL = Crop Land, BShL = Bush land, WL = woody land, BL = bare land.

b. **Standardization and weighting the criteria**

The criteria vector maps were converted to raster data formats for standardization. Linear scale transformation was used to standardize the factors, following the FAO standard for land evaluation for rain-fed agriculture [36]. The factors were categorized into four categories namely highly suitable, moderately suitable, marginally suitable, and not suitable using value ranging from 1 to 4. The weighted factor was determined using the pairwise comparison matrix and AHP matrix with values ranging from 1 to 9 assigned to each factor [37]. The weighting was based on literature reviews, the real nature of the study area, and guidelines from [5]. The purpose of weighting was to express the importance of each factor relative to others in crop yield and growth rate. The priority weights were calculated using the pairwise comparison matrix and eigenvector values.


$$\begin{array}{c} \text{eigenvector}=\text{Aji}=\frac{\sum_{i=1}^{n}{\left(\frac{w1}{w1}*\frac{w1}{w2}*\ldots *\frac{w1}{wn}\right)}^{\frac{1}{n}}}{\Sigma \left[\sum_{i=1}^{n}{\left(\frac{w1}{w1}*\frac{w1}{w2}*\ldots *\frac{w1}{wn}\right)}^{\frac{1}{n}}\right]} \end{array}$$ (1)

Where w1 is the sum of rows for pairwise comparison and n is the size of the matrix

The consistency ratio (CR) was calculated to verify the consistency of comparison as (Table 4).

**Table 4:** Matrix size and Random consistency index.

Matrix size	1	2	3	4	5	6	7	8	9	10	11	12	13	14	15
Random consistency index(RI)	0	0	0.58	0.9	1.12	1.24	1	1.41	1.45	1.49	1.51	1.48	1.56	1.57	1.59

Random Index [27].


$$\text{CI}=\frac{\left(\lambda max-n\right)}{n-1}$$ (2)

Where CI is the consistency index, n is the number of elements being compared in the matrix, and λmax is the largest or principal eigenvalue of the matrix.


$$\text{CR}=\frac{CI}{RI}$$ (3)

Where CR is the consistency ratio, CI is the consistency index, RI is the random index.

If the CR ≤ 0.10, it means the pairwise comparison matrix has a suitable consistency. Otherwise, If CR ≥ 0.10 it implies that pairwise consistency has inadequate consistency [38]. Following the standards weight, a weighted overlay technique was applied to arc map 10.3 to generate suitability maps for each land use type (Equation 4), and a vector overlay analysis was performed to create a composite suitable land allocation map:


$$\begin{array}{c} \text{s}=\sum {\mathrm{\backslash nolimits}}_{i=0}^{n}(WiXi) \end{array}$$ (4)

Where S is the suitability, Wi is the weight of factor i and Xi is the criterion score of factor i.

#### Method of data analysis.

Soil data, including depth, drainage, texture, organic matter, and pH, were compiled and imported into ArcGIS 10.3 for visualization and analysis. Rainfall and temperature data in Excel were examined for missing values and estimated using neighboring stations. Raster images were created from the input data in ArcGIS, and point data were interpolated using the Inverse Distance Weighting tool. An elevation map was derived from a 30 m resolution Digital Elevation Model (DEM), and slope calculations were performed using the Spatial Analyst tool in ArcGIS.

For land use and land cover analysis, Sentinel-2A imagery from 2021 was pre-processed with ERDAS IMAGINE 2015, where images were layer stacked and classified using both supervised and unsupervised methods. Unsupervised classification identified clusters and the number of groups, while supervised classification refined these definitions for thematic maps. The classified images were validated with 240 random points using Google Earth imagery. Satellite images were sourced from USGS Earth Explorer (https://earthexplorer.usgs.gov/), which were downloaded [38]. With image processing and interpretation for land cover mapping conducted by Rwanga and Ndambuki [39].

**Classification of images:** Both supervised and unsupervised classification techniques were used to categorize the pre-processed photos. Using user-provided training sets (signatures) and field-collected ground truth data, the maximum likelihood algorithm is used in the supervised classification technique to classify the image. For the supervised classification, 240 validation or random points (signatures) were utilized for each land use type and land cover. Consequently, the study area’s land cover classes were determined. Land covers change detection analysis.

A base map of the study areas was created using Google Earth satellite imagery and ground truth data. Several features in the study area were identified by manually combining satellite images. The area coverage of the first year was subtracted from that of the second year, as indicated in Equation (1) following Islam [40], to determine the magnitude of change for each LULC class. The difference between the final year and the initial year, which indicates the magnitude of change between corresponding years, was divided by the number of study years to obtain the annual rate of change for each LULC.

**Accuracy assessment:** ArcGIS was used to evaluate the accuracy of the supervised land use classification for the 2021 image. 240 randomly generated points for 2021 supervised and unsupervised images were obtained from the classifier. When the data sets were trained during supervised land use classification, the software itself was able to identify the unique colour tone and pixel value of each and every point. These figures served as the standard values. The user then identified each point that was generated at random and assigned it to a different class. The points that were correctly identified were taken into account for classification. This reference and classified data were also used to create an error matrix and Kappa statistics. The overall accuracy was determined from the error matrix by dividing the total number of examined pixels by the sum of the entries that form the major diagonal. The following equations were also used to calculate the Kappa coefficient of agreement [41].

**Accuracy assessment:** To assess the accuracy of the output map and determine if it meets the required standards, an accuracy assessment was conducted [42–45]. The purpose of this assessment was to evaluate quantitatively how effectively the pixels were classified into the correct land cover classes within the study area. It is recommended by Congalton [46] to have a minimum of 30 sample points per land use/land cover class for accuracy assessment. However, collecting reference samples, especially through ground surveys can be costly. In this study, a minimum of 30 samples were collected for each land use/land cover class to assess the accuracy of the land use/land cover classification. To express the classification accuracy, the most common method used is the error matrix also known as the confusion matrix as described by Lillesand et al. [47]. This matrix provides a cross-tabulation of the classified land cover and the actual land cover determined through the sample site results [45].

**Kappa coefficient:** Kappa coefficient is a measure of overall agreement of a matrix and it is the ratio of the sum of diagonal values to total number of cell counts in the matrix. It describes the proportionate reduction in error generated by the classification process compared with the error of completely random classifications. Kappa values are also characterized into 3 groups: a value greater than 0.75 (75%) represents strong agreement defined as excellent, a value between 0.4 and 0.7 (40–70%) represents moderate agreement (good classification) and a value less than 0.4 (40%) represents poor agreement [48].


$$K=\frac{N\sum_{i=1}^{r}xii-\sum_{i=1}^{r}\left(xi+*x+i\right)}{N^{2}-\sum_{i=1}^{r}\left(xi+*xi+\right)}$$

Where,

K = kappa coefficient

r = number of columns and rows in error matrix,

N = total number of observations,

Xii=observation in column i and row i,

Xi+=marginal total of row i, and

X+i=marginal total of column i.

**Fig 2:**
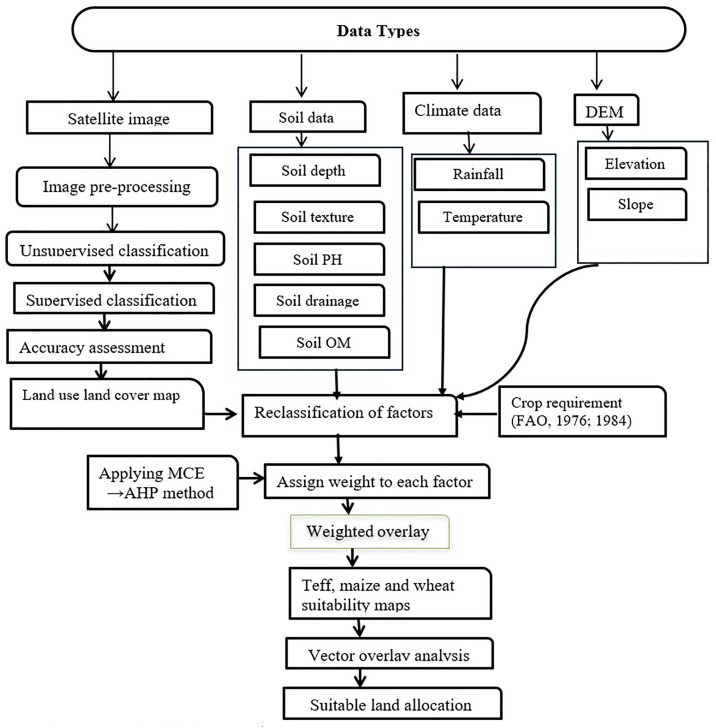
Conceptual framework of the study.

In general, the methodology of this study can be summarized in the following conceptual framework (Fig 2).

### Ethics statement

To get data for this study, IRB or ethics committee who approved or waived the study was not formed. Rather our institution, Arba Minch University wrote a letter to woreda where the Mansa watershed exists. Then the woreda assigned experts to link the researchers with concerned bodies in the study watershed.

## Results and discussions

### Physical factors influencing the suitability of major cereal crops

The soil pH analysis conducted in this study revealed that the soil in the study area is highly acidic with pH values ranging from 3.08 to 5.33. The reclassified soil pH suitability assessment showed that only 29.50% of the watershed is moderately suitable for teff, wheat, and maize crops. About 41.30% of the watershed is marginally suitable, while 29.2% is not suitable for these crops (Table 5). The results indicate that soil pH is a limiting factor for the cultivation of the selected cereal crops in the study area, given the high acidity of the soil. This finding aligns with previous studies by Fekadu and Negese [49] who identified soil pH as a primary limiting factor in the Yikalo sub-watershed. Similarly, Girma et al. [50] found that soil pH and fertility were limiting factors for wheat and maize cultivation in the Jello Watershed. However, the area in which moderately suitable with pH value was larger area than finding by Alemayehu [51] for wheat production in Sinana Research Site of Southeastern Ethiopia. Yohannes and Soromessa [42] identified texture, temperature, slope, and erosion hazard as the main limiting factors for wheat and barley production. However, Girmay et al. [52] discovered that the soil pH in the Gateno watershed has potential for wheat, barley, and faba bean crops.

**Table 5:** Spatial variation of soil pH.

Criteria	Class	Suitability level	Value	Area (km^2^)	Area (%)	Crop type
Soil pH	5.2-5.5	Moderately suitable	2	302.15	29.50	Teff, wheat and maize
	5.0-5.2	Marginally suitable	3	423.01	41.30	Teff, wheat and maize
	<4.5	Not suitable	4	299.08	29.20	Teff, wheat and maize

**Fig 3:**
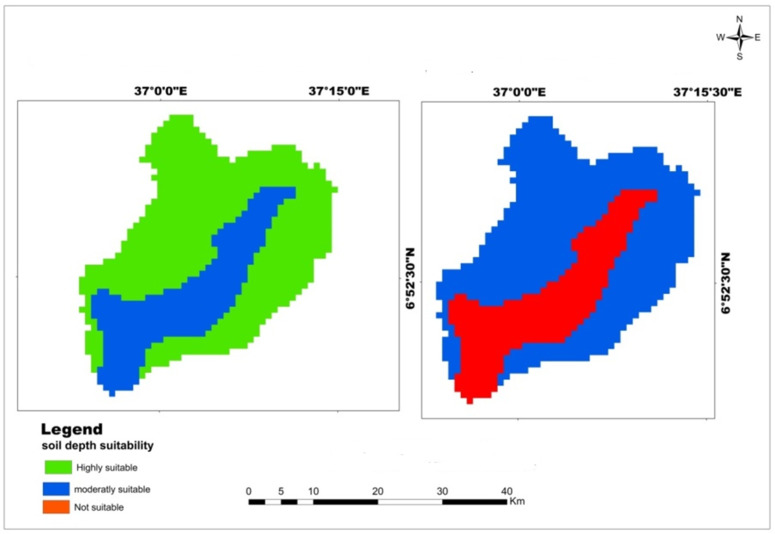
Land suitability map of classified soil depth for teff, maize, and wheat.

The soil depth within the watershed ranges from 34 to 100 cm. The reclassified soil depth suitability analysis showed that 70.33% of the area is highly and moderately suitable for teff and maize crops. Specifically, 70.33% of the watershed has moderate suitability for wheat, and the remaining 29.67% is not suitable (Table 6). The highly and moderately suitable soil depths for teff and maize crops were located in Northwest and Northeast of Mansa Watershed, but not suitable lands were exited in the center of the watershed (Fig 3). The analysis indicates that the study area has potential for teff and maize cultivation in terms of soil depth, but there are limitations for wheat crops. This finding aligns with the research by Yohannes and Soromessa [42], who identified soil depth as a primary limiting factor for wheat and barley cultivation in the Andit Tid watershed. Similarly, Selassie et al. [53] reported that shallow soil depth and low water holding capacity influenced maize production in the Yigossa Watershed. However, the study by Motuma et al. [54] in Wogdie Woreda found that most of the study area had a high suitability in terms of soil depth for wheat and sorghum crops. However, in this study, the area moderately suitable for wheat production was larger area than study by Alemayehu [51] for wheat production in Sinana Research Site of Southeastern Ethiopia.

**Table 6:** Spatial variation of soil depth.

Criteria	Class (cm)	Suitability level	Value	Area (km^2^)	Area (%)	Crop type
Soil Depth	>50	Highly suitable	1	720.35	70.33	Teff and Maize
	34–50	Moderately suitable	2	303.89	29.67	
	75–100	Moderately suitable	2	720.35	70.33	Wheat
	<50	Not suitable	4	303.89	29.67	

**Fig 4:**
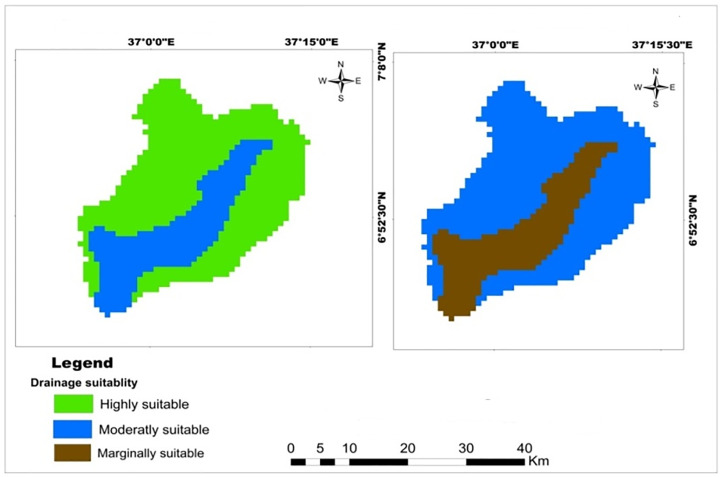
Land suitability map of classified soil drainage for teff, maize, and wheat.

The watershed drainage classes are as follows moderately well-drained and imperfectly drained. The watershed’s reclassified soil drainage map showed that 70.33% and 29.67% of the watershed is highly and moderately suitable for teff and about 70.33% of the watershed is moderately suitable for wheat and maize and 29.67% of the watershed is marginally suitable for wheat and maize crops (Table 7). The highly and moderately suitable soil drainage for teff and maize crops were located in Northwest and Northeast of Mansa Watershed, but marginaly suitable lands were exited in the center of the watershed (Fig 4). The analysis result showed that most part of the watershed has a potential for the cultivation of each selected cereal crop. This result was more consistent with the finding of Mosisa et al. [55] showed that most part of the watershed has good potential for maize crop production. Similarly Girmay et al. [52] found that the soil drainage of Gateno watershed has potential for production of wheat, barley and faba bean crop. However, the area of soil in Mansa watershed had less drained for wheat production than that of area of wheat production in Sinana Research Site of Southeastern Ethiopia [51].

**Table 7:** Spatial variation of soil drainage.

Criteria	Class	Suitability level	Value	Area (km^2^)	Area (%)	Crop type
Soil Drainage	Moderately well drainage	Highly suitable	1	720.35	70.33	Teff
	Imperfectly drainage	Moderately suitable	2	303.89	29.67	
	Moderately well drainage	Moderately suitable	2	720.35	70.33	Wheat and maize
	Imperfectly drainage	Marginally suitable	3	303.89	29.67	

**Fig 5:**
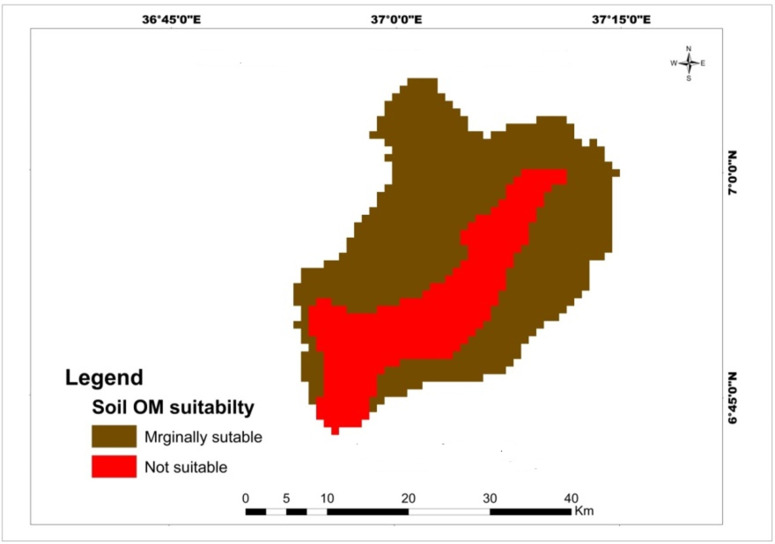
Land suitability map of classified soil organic matter for teff, maize, and wheat.

In Mansa watershed soil organic matter content ranges from 0.223 to 1. 3307. Results for soil organic matter showed that 70.33% and 29.67% of the watershed is marginally suitable and unsuitable for each crop, respectively (Table 8). The majority of marginally suitable soil organic matter content ranges for teff and maize crops was located in Northwest and Northeast of Mansa Watershed, but not suitable lands were exited in the center of the watershed (Fig 5). The required soil organic matter for teff, maize and wheat can be grow successfully in soils with OM content greater than 3% but the watershed soil organic matter is less than 2%. This indicates that soil organic matter is one of the determinant factors in the study area. In line with this finding, soil organic was the main limiting factor for the production of maize in Abobo of western Ethiopia [56]. Similarly, Selassie et al. [53] reported that the main suitability limitations for cereal crops were low organic matter contents and acidic soil pH in Yigossa watershed. Bahir et al. [37] also found that soil organic matter was the main limiting factors in selecting land suitability in Gerado catchment, North-Eastern Ethiopia for wheat and maize production.

**Table 8:** Spatial variation of soil organic matter.

Criteria	Class	Suitability level	Value	Area (km^2^)	Area (%)	Crop type
Soil OM	1–2	Marginally suitable	3	720.35	70.33	Teff, wheat and maize
	<1	Not suitable	4	303.89	29.67	Teff, wheat and maize

**Fig 6:**
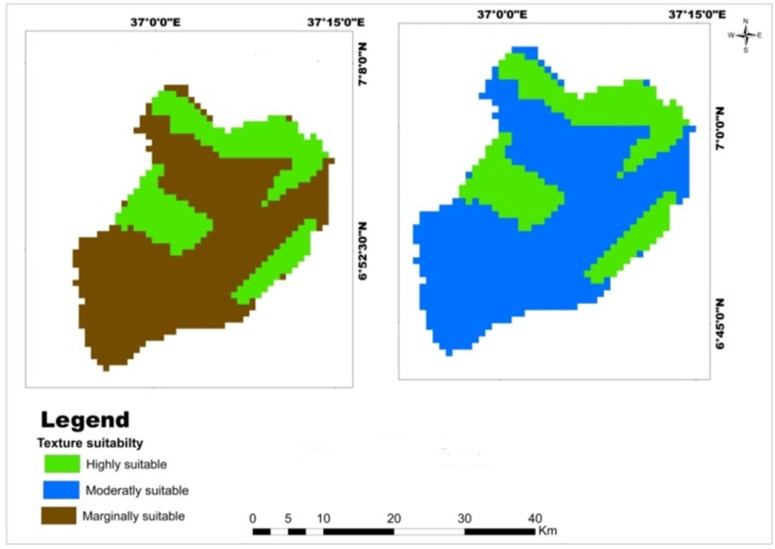
Land suitability map of classified soil texture for teff, maize, and wheat.

The Mansa watershed is predominantly characterized by clay and clay loam soil textures. A reclassification of the soil texture data was conducted to determine the suitability of these textures for the selected cereal crops. The reclassification resulted in three classes. The reclassified soil texture map of the watershed reveals that approximately 29.92% of the area is highly suitable for wheat, teff, and maize, while 70.08% is moderately suitable for wheat but only marginally suitable for teff and maize (Table 9). The majority of marginally and moderately suitable soil textures content ranges for teff and maize crops were located in the south and center of Mansa Watershed, but highly suitable lands were exited in the west and north of the watershed (Fig 6). The findings indicate that the soil textural classes in the study area are favorable for wheat crop production, but there are limitations for teff and maize crops. These findings are consistent with the prior studies conducted by Hussien et al. [57] and Mosisa et al. [55], which also suggested that the soil textures in the study area are moderately appropriate for crop production. Moreover, Motuma et al. [54] found that the soil texture has good potential for the production of wheat and sorghum crops, further supporting the suitability of the soil textures for wheat cultivation in the Mansa watershed.

**Table 9:** Spatial variation of soil texture.

Criteria	Class	Suitability level	Value	Area (km^2^)	Area (%)	Crop type
Soil texture	Clay	Highly suitable	1	306.45	29.92	Wheat
	Clay loam	Moderately suitable	2	717.79	70.08	
	Clay	Highly suitable	1	306.45	29.92	Teff and maize
	Clay loam	Marginally suitable	3	717.79	70.08	

**Fig 7:**
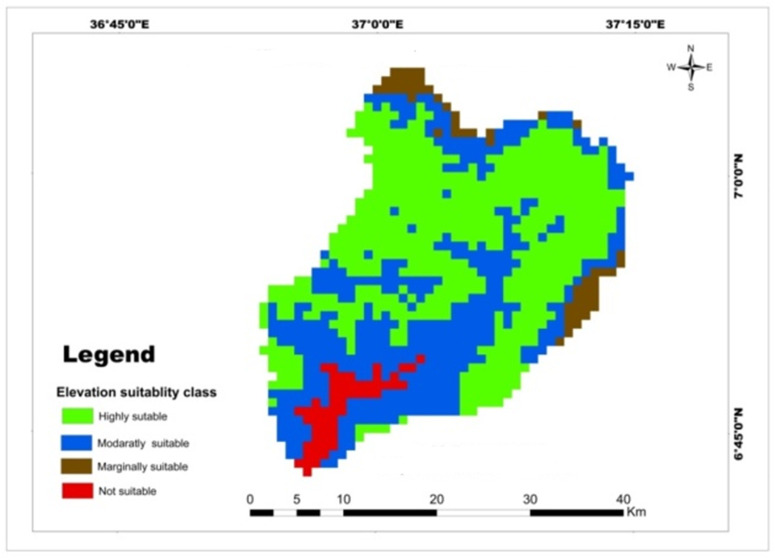
Land suitability map of classified elevation for teff, maize, and wheat.

The elevation map of the watershed was reclassified into four classes to assess its suitability for the selected major cereal crops. The reclassified elevation map reveals that 52.59% of the watershed is highly suitable, 37.21% is moderately suitable, 6.43% is marginally suitable, and 3.76% is not suitable for the selected crops (Table 10). The majority of highly suitable elevations of the lands for teff and maize crops was located in the west, center, and east of the watershed, but moderately suitable lands were exited in the north and east and not suitable lands were in the south of the watershed (Fig 7).The analysis indicates that a significant portion of the watershed has the potential for the production of all selected cereal crops. This finding aligns with the study conducted by Moissa et al. [55], which found that the elevation of the Dedissa watershed was highly suitable for maize cultivation. However, other studies by [49] and Debisa et al. [58] suggested that elevation plays a crucial role in determining cereal crop production. Overall, the reclassified elevation map demonstrates that the majority of the Mansa watershed has favorable conditions for the cultivation of the selected cereal crops

**Table 10:** Spatial variation of elevation.

Criteria	Class	Suitability level	Value	Area (km^2^)	Area (%)	Crop type
Elevation	1600–2200	High suitable	1	538.65	52.59	Teff, wheat and maize
	1000–1600/ 2200–2400	Moderately suitable	2	381.12	37.21	Teff, wheat and maize
	2400–2763	Marginally suitable	3	65.86	6.43	Teff, wheat and maize
	607–1000	Not suitable	4	38.51	3.76	Teff, wheat and maize

**Fig 8:**
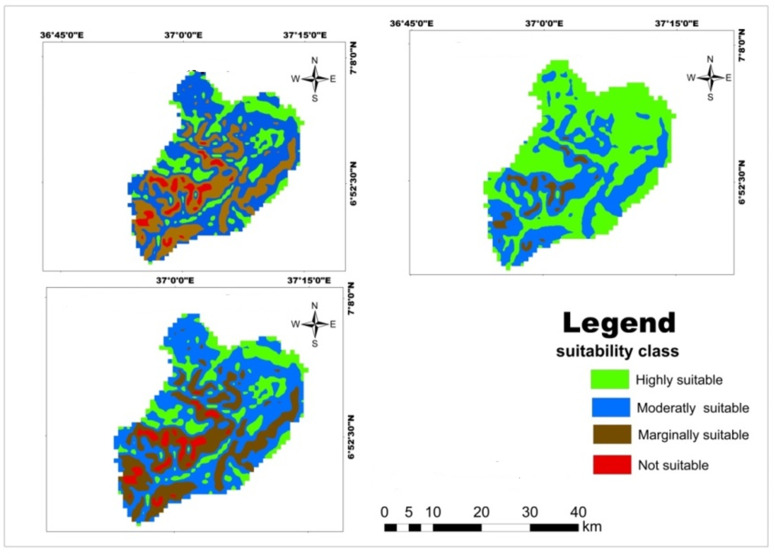
Land suitability map of classified slope for teff, maize, and wheat crops.

The slope of the Mansa watershed ranges from 0.07 to 35.85%. To assess its suitability for the selected cereal crops, the slope values were reclassified, resulting in a slope suitability map for the study area. The reclassified slope map indicates that 19.45% of the area is highly suitable, 48.40% is moderately suitable, 27.89% is marginally suitable, and 4.26% is not suitable for teff and maize crops. For wheat, 57.51% of the area is highly suitable, 38.22% is moderately suitable, and 4.26% is marginally suitable (Table 11). The majority of highly suitable slope ranges for teff and maize crops was located in the north of Mansa Watershed, but not suitable lands were found in the south of the watershed (Fig 8). The analysis reveals that slope is a limiting factor for teff and maize production in the study area, with only a small portion being highly suitable. However, the majority of the watershed is highly suitable for wheat crop production. This finding is consistent with the study conducted by Kahsay et al. [59], which identified slope as a major constraint for rain-fed teff crop production in the degraded semi-arid highlands of Northern Ethiopia. Mulugeta [60] also found that wheat and maize yields are restricted to steep slope locations with shallow soil depth coverage, which are unfavorable for both crops in Ethiopia’s Legambo woreda [61]. Similarly, Rabia [62] identified slope as a limiting factor for teff cultivation in the Kilte Awulaelo district. This result aligns with a study by Esa and Assen [36], which highlighted the significant influence of slope on teff production in the Northwest highlands of Ethiopia. Hence, the analysis demonstrates that slope plays a crucial role in determining the suitability of the Mansa watershed for the selected cereal crops, with wheat being more compatible with the existing slope conditions compared to teff and maize. In our study, the production of wheat was highly suitable at lower slope class of the watershed than upper slopes which was comparable to finding by Alemayehu [51] in Sinana Research Site of Southeastern Ethiopia.

**Table 11:** Spatial variation of slope.

Criteria	Class (%)	Suitability level	Value	Area (km^2^)	Area (%)	Crop type
Slope	0–7	High suitable	1	199.22	19.45	Teff and maize
	7–15	Moderately suitable	2	495.73	48.40	
	15–25	Marginally suitable	3	285.66	27.89	
	>25	Not suitable	4	43.63	4.26	
	0–13	High suitable	1	589.04	57.51	Wheat
	13–25	Moderately suitable	2	391.46	38.22	
	25–35.7	Marginally suitable	3	43.63	4.26	

**Fig 9:**
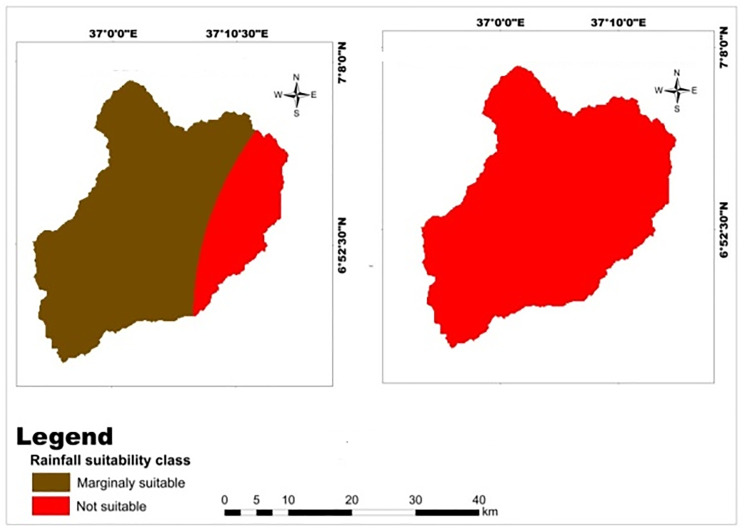
Land suitability map of classified rainfall for teff, maize, and wheat crops.

The precipitation value is reclassified into suitability class for selected cereal crops. The rainfall variation of the study area showed that 79.6% of the area is marginally suitable for maize, but 100% of the area is not suitable for teff and wheat crops (Table 12). The majority of marginally suitable rainfall ranges for teff and maize crops was located in the east and center of Mansa Watershed, but not suitable lands were found in all parts of the watershed (Fig 9). Similarly Mosisa et al. [56] were reported that rainfall of the Dedissa watershed was unsuitable for maize production. The study conducted by Girmay et al. [53] in Gateno watershed showed that rainfall and temperature were the main limiting factor for the production of wheat and barley crops.

**Table 12:** Spatial variation of rainfall.

Criteria	Class (mm)	Suitability level	Value	Area (km^2^)	Area (%)	Crop type
Rainfall	1529–1600	Marginally Suitable	3	815.599	79.6386	Maize
	>1600	Not suitable	4	208.526	20.3614	
Rainfall	1529–1663	Not suitable	4	1024.23	100.0	Teff and Wheat

**Fig 10:**
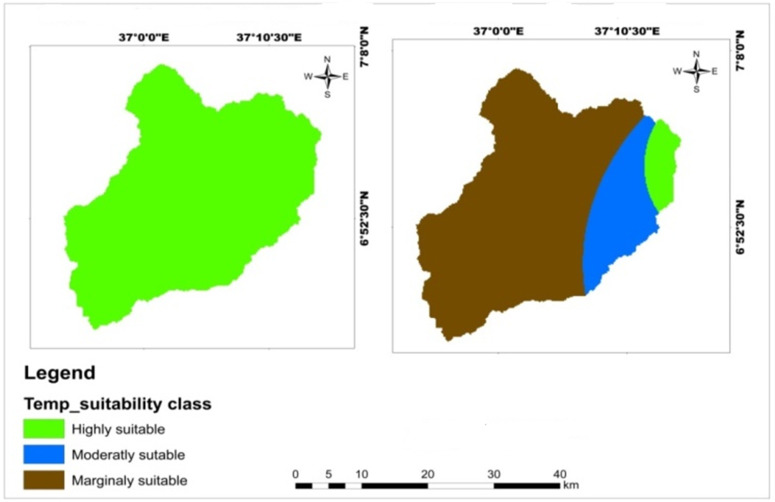
Land suitability map of classified temperature for teff, maize, and wheat crops.

The Mansa watershed experiences an annual average temperature ranging from 16.4°C to 20.5°C. The reclassified temperature map indicates that 5.38% of the watershed is highly suitable, 16.87% is moderately suitable, and 77.77% is marginally suitable for wheat crops (Table 13).The majority of marginally suitable temperature ranges for teff and maize crops was located in center and eastern parts of Mansa Watershed, but a small parcel of highly suitable lands were found in east most of the watershed (Fig 10). This suggests that temperature is the main limiting factor for wheat crop production in the study area. The optimal temperature range required for wheat crops is 14.9–18.4°C, which falls within the highly suitable category. However, the majority of the study area is classified as marginally suitable for wheat cultivation. This finding is consistent with a study by Hailu et al. [63], which identified temperature as the main limiting factor for wheat and barley crops in the Kabe sub watershed. On the other hand, the Mansa watershed is highly suitable for teff and maize crops, as indicated by the reclassified temperature map (Table 14; Fig 13). The analysis suggests that the temperature conditions in the study area are favorable for teff and maize production. However, a study by Yohannes and Soromessa [42] reported that temperature was the most limiting factor for cereal crop production, which contrasts with the findings of this study. Therefore, the analysis reveals that temperature plays a significant role in determining the suitability of the Mansa watershed for wheat, teff, and maize crops. While the temperature conditions in the study area are not optimal for wheat cultivation, they are suitable for teff and maize production.

**Table 13:** Spatial variation of temperature.

Criteria	Class (°C)	Suitability level	Value	Area (km^2^)	Area (%)	Crop type
Temperature	16.4–18.4	Highly suitable	1	54.95	5.38	Wheat
	18.4–19.4	Moderately suitable	2	172.75	16.87	
	19.4–20.5	Marginally suitable	3	796.39	77.77	
	16–20.5	Highly suitable	1	1024.23	100.0	Teff and maize

**Table 14:** LULC types of the study area.

LULC class	Area (km^2^)	%
Forest land	395.11	38.5772
Shrubs cover areas	24.38	2.380011
Grassland	123.23	12.03149
Cropland	480.64	46.92819
Vegetation cover	0.06	0.005595
Bare land	0.11	0.011123
Settlement area	0.68	0.06631
Open water	0.001	0.000287
Total	1024.23	100.0

**Fig 11:**
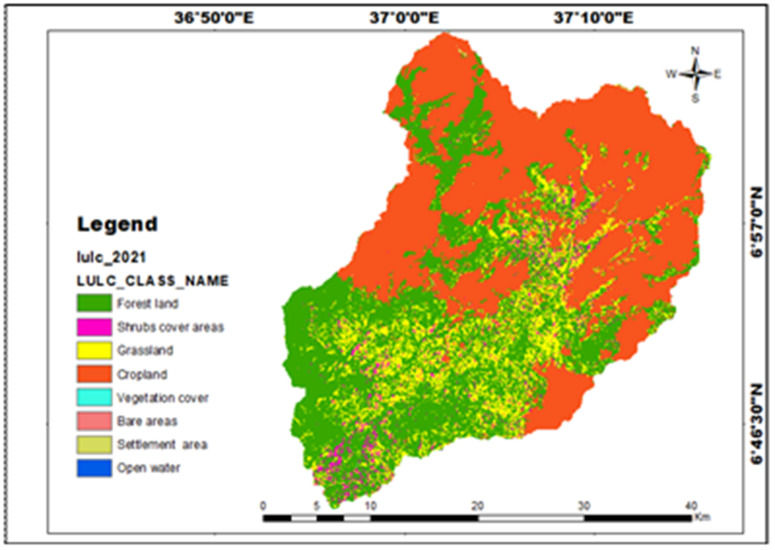
LULC types and their area proportions for Mansa Watershed.

**Fig 12:**
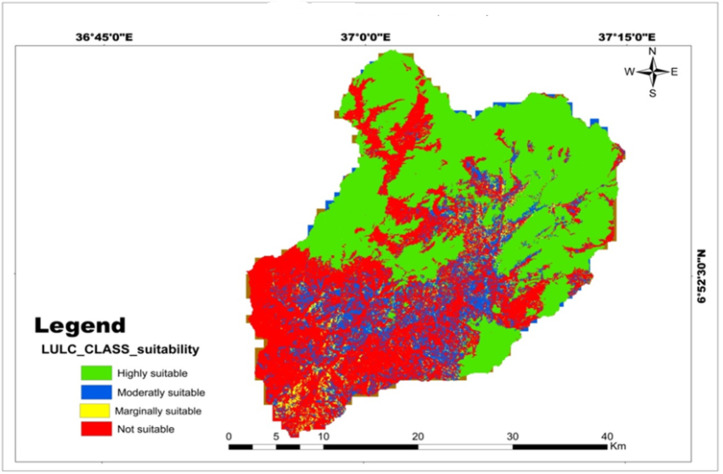
Land suitability map of classified LULC for teff, maize, and wheat crops.

#### Land use/land cover.

For each land use and land cover class, 30 signatures were collected, and the study analysis classified the Sentinel-2A satellite image into eight land cover types: cropland, forest land, grassland, vegetation covers, shrub covers, bare land, settlement area, and open water. The maximum likelihood classification of the image revealed that cropland occupies the largest portion of the area in the northern parts of the watershed (Fig 11) which were highly suitable for production for the selected crops for these analyses (Fig 12), while water bodies cover a relatively small area compared to the other land cover types (Table 14).

#### Accuracy assessment of LULC classification.

The accuracy assessment of the classification resulted in an overall accuracy of 88.7% and a kappa coefficient of 0.87 (Table 15). These values indicate a high level of accuracy and reliability in the classification results, providing a solid foundation for further analysis. The satisfactory classification accuracy assessment results align with previous studies [46,47], further validating the findings. The land use and land cover (LULC) types play a crucial role in determining the adaptability of various important cereal crops. Therefore, the LULC of the watershed was categorized into four groups based on crop suitability, taking into account the impact of LULC on the growth and productivity of these crops (Table 16; Fig 12).

**Table 15:** Accuracy assessment.

Reference Data										
Classified data	Forest land	Shrubs cover areas	Grassland	Cropland	Vegetation cover	Bare land	Settlement areas	Open water	Total	User accuracy
Forest land	27	1	1	0	2	0	0	0	31	87.1
Shrubs cover areas	2	28	1	0	0	0	0	0	31	90.3
Grassland	0	1	27	2	0	0	0	0	30	90.0
Cropland	0	0	1	26	0	6	1	0	34	76.5
Vegetation cover	1	0	0	0	28	0	0	0	29	96.6
Bare land	0	0	0	2	0	22	4	0	28	78.6
Settlement area	0	0	0	0	0	2	25	0	27	92.6
Open water	0	0	0	0	0	0	0	30	30	100.0
Total	30	30	30	30	30	30	30	30	240	
Producer accuracy	90.0	93.3	90.0	86.7	93.3	73.3	83.3	100.0		

**Table 16:** LULC class with respective suitability for the selected crop.

LULC class	Suitability level	Value	Area (km^2^)	Percent
Cropland	High suitable	1	480.64	46.93
Grassland	Moderately suitable	2	123.22	12.03
Shrubs cover areas/bare areas	Marginally suitable	3	24.49	2.39
Forest land/vegetation/settlement areas/open water	Not suitable	4	395.85	38.65
	Total		1024.23	100.0

### Physical land suitability analysis results for selected major cereal crops

**Fig 13:**
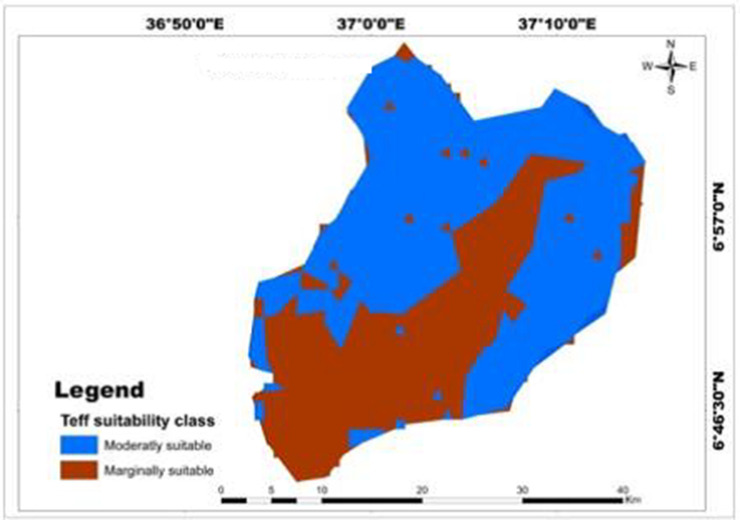
Land suitability map classified soil depth for teff.

**Fig 14:**
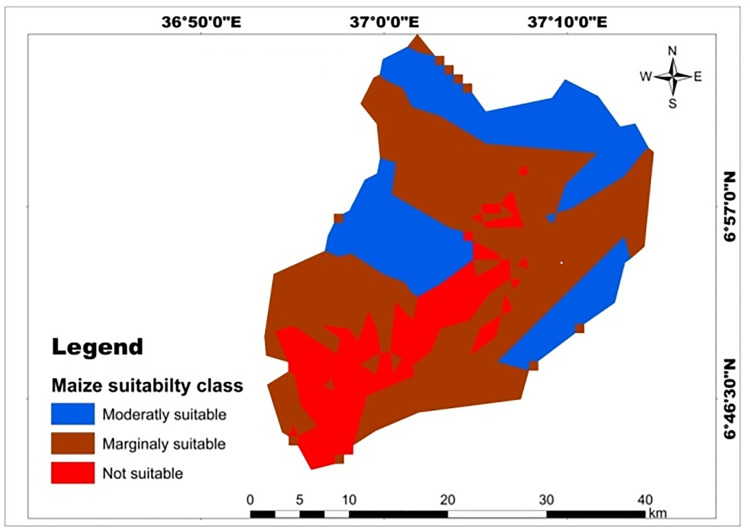
Land suitability map classified soil depth for maize crop.

**Fig 15:**
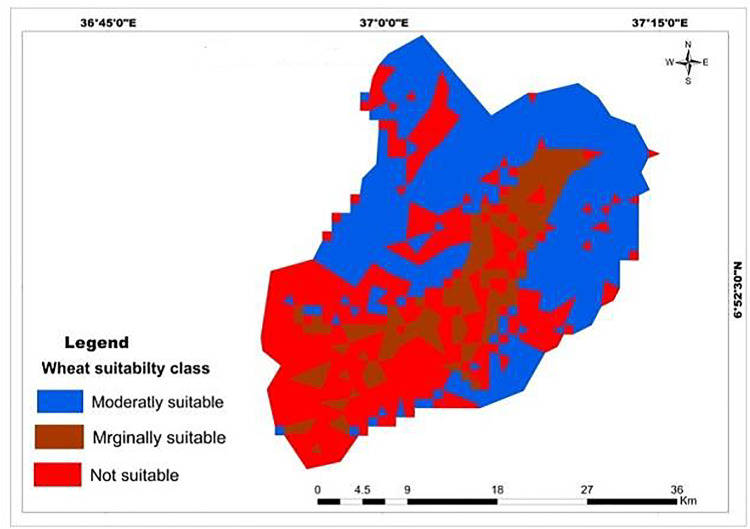
Land suitability map classified soil depth for wheat crop.

Results of a physical land suitability analysis for teff, maize, and wheat crops were obtained through a weighted overlay analysis of ten factors. The analysis revealed that in the study area, 61.18% (626.5 km^2^) and 38.82% (397.52 km^2^) of the land were moderately suitable and marginally suitable for teff crops respectively (Table 17; Fig 13). The majority of highly suitable LULC type for teff and maize crops was located in the north and east of Mansa Watershed, but not suitable lands were found in the south and southwest of the watershed Figs 13,14). However, no areas were classified as highly suitable (S1) or not suitable (N1) for teff cultivation. This indicates that the study area has significant limitations in terms of rainfall, soil organic matter, pH, soil texture, elevation, soil drainage, and soil depth. These findings align with previous research conducted in Northern Ethiopia’s degraded semi-arid highlands by Kahsay et al. [59] and the Gelda catchment by Esa and Assen [36]. However, a different study in Kilte Awulaelo Woreda, Tigray region showed that most of the study area was permanently unsuitable for teff and maize production Hishe and Assen [64]. For wheat production, 50.063% (512.703 km^2^), 13.9% (142.398 km^2^), and 36.03% (368.99 km^2^) of the land in the study area were assessed as moderately suitable, marginally suitable, and not suitable, respectively (Table 17; Fig 15). Similar findings were reported in South Wello, Yikalo sub-watershed, Kabe sub-watershed, Andit Tid watershed, Anjeni watershed [65], and Awulaelo District in Tigray region (Motuma et al. [54], Fekadu and Negese [49], Hailu et al. [63], Yohannes and Soromessa [42], Rabia [62]). These studies identified varying proportions of moderately suitable, marginally suitable, and highly suitable areas for wheat production. Regarding maize cultivation, 29.65% (303.62 km^2^), 52.81% (540.78 km^2^), and 17.55% (179.72 km^2^) of the land in the study area were classified as moderately suitable, marginally suitable, and not suitable, respectively (Table 17; Fig 14). Similar findings were reported in the Dedissa watershed, Bilate Alaba Sub-watershed, Abobo region of western Ethiopia, and Dabo Hana district, indicating varying proportions of suitability for maize crop production [4,53,56,58]. Overall, the results of the analysis demonstrate the suitability of the study area for teff, wheat, and maize cultivation, but also highlight the need for addressing the limitations identified through sustainable land management strategies.

**Table 17:** Area coverage of land suitability for selected cereal crops in the watershed.

Suitability Classes	Maize		Teff		Wheat	
	Area(km^2^)	(%)	Area(km^2^)	(%)	Area(km^2^)	(%)
S2	303.62	29.65	626.50	61.18	512.703	50.063
S3	540.78	52.81	397.52	38.82	142.398	13.9
N1	179.72	17.55	–	–	368.99	36.03

### Land allocation for major cereal crops using current suitability maps

**Fig 16:**
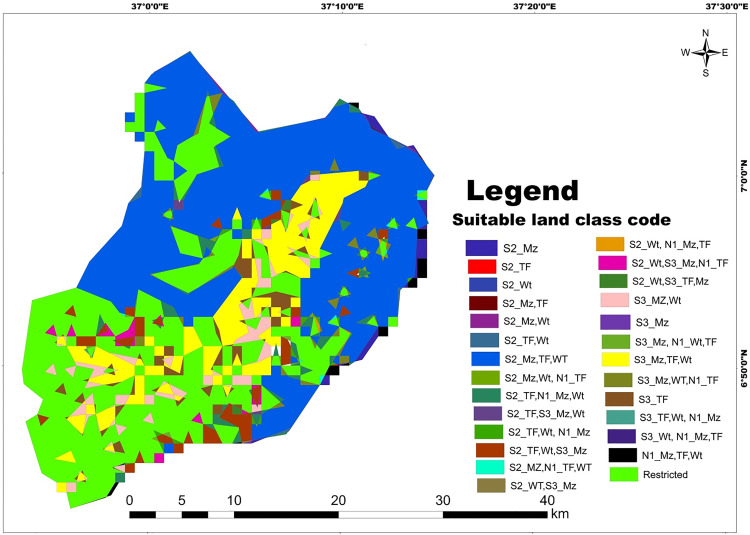
Appropriate Land Allocation Map with their Respective Degree of Suitability in Mansa watershed (2022) (Note: S2 = moderately suitable, S3 = marginally suitable, N1 = currently not suitable, Tf = Teff, Mz = Maize, Wt = Wheat).

The suitable land allocation for selected land use types (LUTs) in the watershed, based on their best suitability classes is presented in Table 18. Approximately 44.2% (442.1 km^2^) and 10.1% (110.2 km^2^) of the study area are classified as moderately suitable (S2) and marginally suitable (S3) for all specified land use categories, respectively (Fig 16). This finding aligns with similar studies conducted in other regions [25,65], indicating that different LUTs can compete for the same land with equal suitability ratings. The remaining area exhibits mixed suitability for the analyzed crops. For instance, about 2.7% (27.9 km^2^) of land is moderately suitable (S2) for teff and wheat production but only marginally suitable (S3) for maize crops. Similarly, approximately 0.51% (1.53 km^2^) of land is marginally suitable (S3) for maize and teff production but moderately suitable (S2) for wheat cultivation. This indicates that farmers have the flexibility to choose land use types that best suit their requirements [66]. However, these findings also highlight the competition for land among various land use alternatives with different suitability levels [58,60]. The remaining suitable land allocation classes represent small patches of land across the study area (Table 18). In the future work, the production of these crops could integrate socioeconomic factors like market and irrigation facilities in the study area which are vital for agricultural planning [67].

**Table 18:** Suitable land allocation and area coverage for selected cereal crops.

Overall Suitability Analysis			
SN	Code	Area (km^2^)	Area (%)
1	S2_Mz	7.87	0.7
2	S2_TF	0.054	0.005
3	S2_Wt	0.39	0.03
4	S2_TF,Wt	2	0.2
5	S2_Mz.TF	0.28	0.02
6	S2_Mz,Wt	1.1	0.1
7	S2_Mz,TF,Wt	442.1	44.2
8	S2_Mz,Wt,N1_TF	1.1	0.1
9	S2_TF,N1_Mz,Wt	18.6	1.8
10	S2_ TF, S3_Mz,Wt	2.1	0.2
11	S2_Wt,TF, N1_Mz	3.8	0.3
12	S2_TF,Wt, S3_Mz	27.9	2.7
13	S2_Mz, N1_Wt,TF	1.35	0.13
14	S2_Wt, S3_Mz	0.13	0.1
15	S2_Wt,N1_Mz,TF	0.7	0.07
16	S2_Wt, S3_Mz, N1_TF	7.1	0.7
17	S2_Wt,S3_TF,Mz	1.53	0.15
18	S3_Mz,Wt	31.9	3.1
19	S3_Mz	0.2	0.02
20	S3_Mz, N1_Wt,TF	1.3	0.13
21	S3_Mz,TF,Wt	110.2	10.1
22	S3_Mz,Wt,N1_TF	10.2	1.02
23	S3_TF	23.3	2.3
24	S3_Wt,TF,N1_Mz	0.19	0.01
25	S3_Wt,N1_TF,Mz	0.12	0.01
26	N1_Mz,TF,Wt	6.2	0.6
27	Restricted	322	32.
	Total	1024.23	100.0

## Conclusions

This study utilized GIS and AHP approaches to assess the suitability of land for selected cereal crops in the Mansa watershed, aiming for sustainable agriculture. The results revealed that there were no highly suitable lands for the production of the selected major cereal crops. Instead, most of the lands in the watershed were classified as moderately and marginally suitable for teff, wheat, and maize crops. The study also identified significant differences in land suitability among different land units for each cereal crop. Teff cultivation was found to be relatively better suited compared to the current land use practices of maize and wheat. However, wheat and maize cultivation still remain viable options. The study identified soil pH, organic matter, and rainfall as the main limiting factors for the production of teff, wheat, and maize crops. Proper management measures are necessary to address these limitations and maintain the productivity of the farmlands.

It was found that there were no highly suitable lands for the production of selected major cereal crops. Instead, most of the lands in the watershed were classified as moderately and marginally suitable. In the future, sustainable land management interventions will be essential for enhancing land suitability and improving soil properties. Additionally, the analysis should encompass irrigation facilities, market access, processing industries, and other socioeconomic factors to offer a wider range of options for stakeholders and policy decision makers. Lastly, appropriate conservation measures should be implemented to maximize crop production and sustain the soil’s productive capacity in areas classified as moderately and marginally suitable for the selected cereal crops.

## References

[1] Mohammad SN, Mohd MA. Land suitability analysis for sustainable agricultural land use planning in Bulandshahr district of Uttar Pradesh. Int J Sci Res Publ. 2014;4:1–11.

[2] AGRA (Alliance for a Green Revolution in Africa). Africa Agriculture Status Report: Focus on Stable crops. Nairobi, Kenya; 2013.

[3] McDowell RW, Snelder T, Harris S, Lilburne L, Larned ST, Scarsbrook M, et al. The land use suitability concept: Introduction and an application of the concept to inform sustainable productivity within environmental constraints. Ecol Indic. 2018;91:212–9. doi: 10.1016/j.ecolind.2018.03.067

[4] Mathewos M, Dananto M, Erkossa T, Mulugeta G. Parametric land suitability assessment for rain fed agriculture: The case of Bilate Alaba sub-watershed, Southern Ethiopia. Agro Technol. 2018;7:183.

[5] Food and Agriculture Organization. A framework for land evaluation: Soils Bulletin 32. Rome, Italy: FAO of the United Nations; 1976.

[6] Tadesse M, Negese A. Land suitability evaluation for sorghum crop by using GIS and AHP techniques in Agamsa sub-watershed, Ethiopia. Cogent Food Agric. 2020;6(1):1743624. doi: 10.1080/23311932.2020.1743624

[7] Pricope NG, Husak G, Lopez-Carr D, Funk C, Michaelsen J. The climate-population nexus in the East African Horn: Emerging degradation trends in rangeland and pastoral livelihood zones. Glob Environ Change. 2013;23(6):1525–41. doi: 10.1016/j.gloenvcha.2013.10.002

[8] CSA (Central Statistics Agency). Ethiopian National Population and Housing Census. Addis Ababa: Central Statistical Agency; 2017. pp. 385.

[9] Pirbalouti A, Ghasemi M, Bahrami A, Golparvar R, Abdollahi K. GIS-based land suitability assessment for German chamomile production. Bulg J Agric Sci. 2011;17:93–8.

[10] Ebrahim E, Mohamed A. A GIS based land suitability analysis for sustainable agricultural planning in Gelda catchment, Northwest Highlands of Ethiopia. J Geogr Reg Plann. 2017;10(5):77–91. doi: 10.5897/jgrp2016.0586

[11] Worqlul AW, Jeong J, Dile YT, Osorio J, Schmitter P, Gerik T, et al. Assessing potential land suitable for surface irrigation using groundwater in Ethiopia. Appl Geogr. 2017;85:1–13.

[12] Taffesse A, Dorosh P, Asrat S. Crop production in Ethiopia: Regional patterns and trends (ESSP II Working Paper 16). Philadelphia USA: International Food Policy Research Institute (IFPRI) in Ethiopia Strategy Support Program (ESSP II), University of Pennsylvania; 2012.

[13] Gadana BD. Determinants and consequences of food insecurity: The case of Loma Bosa District, Dawro Zone. Adv Ecol Environ Res. 2021;6:23–35.

[14] Yohannes H, Soromessa T. Land suitability assessment for major crops by using GIS-based multi-criteria approach in Andit Tid watershed, Ethiopia. Cogent Food Agric. 2018;4(1):1470481. doi: 10.1080/23311932.2018.1470481

[15] Singha C, Swain KC. Land suitability evaluation criteria for agricultural crop selection: a review. Agric Rev. 2016;37(2). doi: 10.18805/ar.v37i2.10737

[16] Ownegh M, Ghanghermeh A, Abedi G. Land use management plan for southeastern coasts of the Caspian Sea: (Introduction a numerical model for ecological potential assessment and land use planning). Agric Nat Resour Sci. 2016;3(15):139–52.

[17] Ray S. A comparative analysis and testing of supervised machine learning algorithms. Vellore, Tamil Nadu, India: School of Electronics Engineering Vellore Institute of Technology; 2018.

[18] Pugliese R, Regondi S, Marini R. Machine learning-based approach: global trends, research directions, and regulatory standpoints. Data Sci Manage. 2021;4:19–29. doi: 10.1016/j.dsm.2021.12.002

[19] Hordofa T, Alemu M, Eshetu M. Land Suitability Analysis and Mapping Using Geospatial Technique and Multi Criteria Decision Analysis for Urban Green Area of Goba Town, Oromia, Ethiopia. Landsc Arch Reg Plann. 2024;9(2):25–37. doi: 10.11648/j.larp.20240902.11

[20] Abel B, Alemu M, Leul Y, Feye T. Suitable landfill site selection using GIS-based multi-criteria decision analysis and evaluation in Robe town, Ethiopia. GeoJournal. 2020;87:895–920.

[21] Al-Djazouli MO, Elmorabiti K, Rahimi A, Amellah O, Fadil OAM. Delineating of groundwater potential zones based on remote sensing, GIS and analytical hierarchical process: a case of Waddai, eastern Chad. GeoJournal. 2020;86(4):1881–94. doi: 10.1007/s10708-020-10160-0

[22] Zhang J, Xu C, Song Z, Huang Y, Wu Y. Decision framework for ocean thermal energy plant site selection from a sustainability perspective: the case of China. J Clean Prod. 2019;225:771–84. doi: 10.1016/j.jclepro.2019.04.032

[23] Christian H, Macwan JE. Landfill site selection through GIS approach for fast growing urban area. Int J Civ Eng Technol. 2017;8(11):10–23.

[24] Malczewski J. GIS-based land-use suitability analysis: a critical overview. Progr Plann. 2004;62(1):3–65. doi: 10.1016/j.progress.2003.09.002

[25] Krügel F, Mas S, Hindorf P, Buthmann E. An online multicriteria—spatial decision support system for public services planning. Appl Sci. 2024;14:1526.

[26] Taherdoost H, Madanchian M. Multicriteria decision making (MCDM) methods and concepts. Encyclopedia. 2023. pp. 77–87.

[27] Gharye MM, Gholami S, Rahmani D. A mathematical model for the optimization of agricultural supply chain under uncertain environmental and financial conditions: the case study of fresh date fruit. Environ Dev Sustain. 2024;26:20807–40.

[28] MWANRO (Maraka Woreda Agricultural and Natural Resource Office). Area description of Mansa Watershed. Southwestern Ethiopia, Tercha; 2021.

[29] Bureau of Finance and Economic Development (BoFED). Annual Statistics abstract of CSA and reported by BoFED of SNNPS. 2019.

[30] Debalke BD, Mengistu AD, Admas ET. Physical land suitability evaluation of rainfed crop production of wheat, barley, and teff in Arsi zone of Ethiopia. Arab J Geosci. 2023;16(177).

[31] Food and Agriculture Organization (FAO). GEONETWORK: Major soil groups of the world (FGGD). Rome: FAO; 2013.

[32] Sys C, Van ranst E, Debaveye J, Beernaert F. Land Evaluation. Part III: Crop Requirements. Brussels, Belgium: General Administration for Development Cooperation; 1993.

[33] Gessesse B, Ali A, Regassa A. Land Evaluation and Land Use Planning. In: Beyene S, Regassa A, Mishra BB, Haile M, editors. The Soils of Ethiopia. World Soils Book Series. Cham: Springer; 2023.

[34] Zech W, Schad P, Hintermaier-Erhard G. Soils of the World. Germany: Verlag GmbH; 2002.

[35] Agidew AA. Land suitability evaluation for sorghum and barley crops in South Wollo Zone of Ethiopia. J Econ Sustain Dev. 2015;6:14–26.

[36] Ebrahim E, Mohamed A. A GIS based land suitability analysis for sustainable agricultural planning in Gelda catchment, Northwest Highlands of Ethiopia. J Geogr Reg Plann. 2017;10(5):77–91. doi: 10.5897/jgrp2016.0586

[37] Bahir AL, Ahmed MA, Antille DL. Land suitability evaluation to optimize land management of small-scale farms in the Gerado catchment, North-Eastern Ethiopia. Trop Agric. 2015;92:49–68.

[38] Rongali G, Kumar KA, Kumar GA, Khosa R. A mono-window algorithm for land surface temperature estimation from Landsat 8 thermal infrared sensor data: a case study of the Beas River Basin, India. Pertanika J Sci Technol. 2018;26(2):829–40.

[39] Rwanga SS, Ndambuki JM. Accuracy assessment of land use/land cover classification using remote sensing and GIS. J Geosci. 2017;08(04):611–22. doi: 10.4236/ijg.2017.84033

[40] Islam K, Jashimuddin M, Nath B, Nath TK. Land use classification and change detection by using multi-temporal remotely sensed imagery: The case of Chunati wildlife sanctuary, Bangladesh. Egypt J Remote Sens Space Sci. 2018;21(1):37–47. doi: 10.1016/j.ejrs.2016.12.005

[41] Afify HA. Evaluation of change detection techniques for monitoring land-cover changes: a case study in new Burg El-Arab area. Alexandria Eng J. 2011;50(2):187–95. doi: 10.1016/j.aej.2011.06.001

[42] Yohannes H, Soromessa T. Land suitability assessment for major crops by using GIS-based multi-criteria approach in Andit Tid watershed, Ethiopia. Cogent Food Agric. 2018;4(1):1470481. doi: 10.1080/23311932.2018.1470481

[43] Malczewski J. GIS-based land-use suitability analysis: a critical overview. Program Plan. 2004;62:3–65.

[44] Saaty TL. The analytic hierarchy process. Planning priority setting, Resource allocates. 1980.

[45] Takala W, Adugna T, Tamam D. Land use land cover change analysis using multi temporal landsat data in gilgel gibe, omo gibe basin, Ethiopia. Int J Sci Technol. 2016;5:309–23.

[46] Congalton RG. Accuracy assessment and validation of remotely sensed and other spatial information. Int J Wildland Fire. 2001;10(4):321. doi: 10.1071/wf01031

[47] Lillesand TM, Kiefer RW, Chapman JW. Remote Sensing and Image Interpretation. 6th ed. New York: Wiley; 2008.

[48] Viera AJ, Garrett JM. Understanding interobserver agreement: the kappa statistic. Fam Med. 2005;37(5):360–3. 15883903

[49] Fekadu E, Negese A. GIS assisted suitability analysis for wheat and barley crops through AHP approach at Yikalo sub-watershed, Ethiopia. Cogent Food Agric. 2020;6(1):1743623. doi: 10.1080/23311932.2020.1743623

[50] Girma R, Moges A, Quraishi S. GIS-based physical land suitability evaluation for crop production in Eastern Ethiopia: A case study in Jello watershed. Agro Technol. 2015; 5:139–45.

[51] Alemayehu A. Land suitability analysis for sustainable production of selected cereals in southeastern Ethiopia. Appl Environ Soil Sci. 2023;2023:1–12. doi: 10.1155/2023/6688187

[52] Girmay G, Sebnie W, Reda Y. Land capability classification and suitability assessment for selected crops in Gateno watershed, Ethiopia. Cogent Food Agric. 2018;4(1):1532863. doi: 10.1080/23311932.2018.1532863

[53] Selassie YG, Ayalew G, Elias E, Getahun M. Soil characterization and land suitability evaluation to cereal crops in Yigossa Watershed, Northwestern Ethiopia. J Agric Sci. 2014;6(5). doi: 10.5539/jas.v6n5p199

[54] Motuma M, Suryabhagavan KV, Balakrishnan M. Land suitability analysis for wheat and sorghum crops in Wogdie District, South Wollo, Ethiopia, using geospatial tools. Appl Geomat. 2016;8(1):57–66. doi: 10.1007/s12518-016-0168-5

[55] Moisa MB, Tiye FS, Dejene IN, Gemeda DO. Land suitability analysis for maize production using geospatial technologies in the Didessa watershed, Ethiopia. Artif Intell Agric. 2022;6:34–46. doi: 10.1016/j.aiia.2022.02.001

[56] Yitbarek T, Kibret K, Gebrekidan H, Beyene S. Physical land suitability evaluation for rain fed production of cotton, maize, upland rice, and sorghum in Abobo area, western Ethiopia. Am J Res Commun. 2013;1:296–318.

[57] Hussien K, Woldu G, Birhanu S. A GIS-based multi-criteria land suitability analysis for surface irrigation along the Erer Watershed, Eastern Hararghe Zone, Ethiopia. East Afr J Sci. 2019;13:169–84.

[58] Debisa G, Gebre SL, Melese A, Regassa A, Teka S. GIS and remote sensing-based physical land suitability analysis for major cereal crops in Dabo Hana district, South-West Ethiopia. Cogent Food Agric. 2020;6.

[59] Kahsay A, Haile M, Gebresamuel G, Mohammed M. GIS-based multi-criteria model for land suitability evaluation of rainfed teff crop production in degraded semi-arid highlands of Northern Ethiopia. Model Earth Syst Environ. 2018;4(4):1467–86. doi: 10.1007/s40808-018-0499-9

[60] Mulugeta H. Land suitability and crop suitability analysis using remote sensing and GIS application; a case study in Legambo woreda, Ethiopia. Ethiopia: Addis Ababa University; 2010.

[61] Bozdağ A, Yavuz F, Günay AS. AHP and GIS based land suitability analysis for Cihanbeyli (Turkey) County. Environ Earth Sci. 2016;75(9). doi: 10.1007/s12665-016-5558-9

[62] Rabia AH, Terribile F. Introducing a New Parametric Concept for Land Suitability Assessment. Int J Enviro Sci Dev. 2013;:15–9. doi: 10.7763/ijesd.2013.v4.295

[63] Hailu AH, Kibret K, Gebrekidan H. Land suitability evaluation for rain-fed production of barley and wheat at Kabe subwatershed, northeastern Ethiopia. Afr J Soil Sci. 2015;3:147–56.

[64] Hishe S, Assen M. GIS based land suitability analysis for selected cereals in five peasant associations of Kilte Awulaelo Woreda, Tigray, Northern Ethiopia. J Agric Environ Sci. 2016;3:10–4.

[65] Alemu WG, Amare T, Yitaferu B, Selassie YG, Wolfgramm B, Hurni H. Impacts of soil and water conservation on land suitability to crops: the case of Anjeni watershed, northwest Ethiopia. J Agric Sci. 2013;5:95–109.

[66] Duguma WD. GIS and remote sensing based land suitability analysis for agricultural crops in Mojo watershed, upper Awash sub basin. Ethiopia: Addis Ababa University; 2010.

[67] Shavanov MV, Magomadov AS, Slavkina VE. Factors affecting arable agriculture in the future. In: IOP Conf. Ser.: Earth Environ. Sci. 2022.

