# Supplementary material for: Analyzing land suitability for key cereal crops in Mansa Watershed, Southwest Ethiopia
Source: PLoS One. 2025 Aug 22;20(8):e0328533. doi: 10.1371/journal.pone.0328533 (PMC12373178; doi:10.1371/journal.pone.0328533)
Supplement: Attachment — Submitted filename: Manuscript.pdf [file pone.0328533.s001.pdf]

Hindawi
Applied and Environmental Soil Science
Volume 2023, Article ID 6688187, 12 pages
https://doi.org/10.1155/2023/6688187

Research Article

Land Suitability Analysis for Sustainable Production of Selected Cereals in Southeastern Ethiopia

Arragaw Alemayehu

Department of Geography and Environmental Studies, Debre Berhan University, Debre Berhan, Ethiopia

Correspondence should be addressed to Arragaw Alemayehu; arragawalex@gmail.com

Received 13 February 2023; Revised 23 August 2023; Accepted 28 August 2023; Published 4 September 2023

Academic Editor: Francesco De Mastro

Copyright © 2023 Arragaw Alemayehu. This is an open access article distributed under the Creative Commons Attribution License, which permits unrestricted use, distribution, and reproduction in any medium, provided the original work is properly cited.

This study has evaluated suitability of the Sinana Research Site (SRS) (southeastern Ethiopia) for sustainable wheat and barley production to improve local food security. Physical land evaluation was undertaken using data about ten land characteristics. Matching land characteristics with land use requirements was made, first. Decision on the overall level of suitability of the area for wheat and barley farming was conducted using the "maximum limiting factor" technique. Over two-third (70.2%) of SRS was marginally suitable for barley farming, but 29.8% of the area was "not suitable" for it. Only slightly less than three-fifth (57.6%) of the area was marginally suitable for wheat production, and the rest 42.4% was "not suitable" for it. Deficient N and P supply and soil alkalinity were critical constraints of land suitability for both crops. Thus, integrated application of inorganic and organic fertilizers and rotating leguminous crops with wheat and barley are useful to mitigate deficiency of N and P in the soil. Hence, farms under "marginally" and "not" suitable classes could be improved to moderate (S2) or high (S1) level of suitability for the crops studied. This, in turn, improves yield and food security status of households in the study area.

1. Introduction

Land suitability evaluation is an assessment of land qualities and characteristics and matching them with the requirements of existing or intended uses; that is, it indicates how well qualities of a "land unit" match the requirements of a particular land use type [1–3]. Land evaluation and land use planning aim to resolve conflicts between competing land uses, the interests of individuals and the community, and conflicting interests between the present and future generations [3]. It depends on soil surveys, which are vital sources of evidences about land qualities and characteristics used for evaluation. The knowledge about soil and its characteristics, spatial distribution, and potentials for different land uses should be given more emphasis for suitability analysis of any crop [4–7]. Since crops require definite environmental conditions for optimum yield, analysis of soil, topography, and climate-based land suitability for specific uses are of great worth [4, 5, 7, 8]. Land suitability analysis, as a tradition, is thought to depend more on land (soil)-related parameters in physical land suitability evaluation [9].

In Ethiopia, population pressure, overcultivation, high soil loss rate, declining soil fertility, overall land degradation, unsustainable farming systems, and climate variability [10–15] are critical threats on yield of crops and hence for the persistence of food insecurity problem in the country [16]. Moreover, declining nutrient storage and moisture retention capacity, change in texture composition, dwindling organic matter supply, increasing acidity, high soil erosion rate, and poor soil fertility are the characteristic features of soils and key productivity constraints of land in highland areas of Ethiopia [4–6, 11, 17, 18]. High fragmentation of land, declining landholding size, little fallowing, and declining land productivity are also vital land use-related problems in highland areas of the country [18, 19]. Because of these and growing cost of food, food insecurity is still a critical problem in Ethiopia [20, 21]. Ethiopia has failed to ensure food self-sufficiency by the end of 2015 as one of the eight Millennium Development Goals (MDGs) targets to have been attained.

Ensuring food security and sustainable food production through resilient farming practices is boldly shown as “Goal 2” in agenda 2030, which requires improving production and yield of land. Increasing crop production for the growing population in the face of climate variability seems difficult in sub-Sahara nations of Africa. Ensuring food security at the household level should begin from tackling climate variability and strengthening capacity for adaptation to climate change [22]. Thus, land suitability evaluations should integrate climate components to make the evaluation complete, improve yield, and ensure food security [8, 23].

Raising crop production is an essential part of ensuring adequate food availability and improving food security in Africa including Ethiopia [16, 24]. Knowledge and evidence-based land use is one way for improving crop yield, which requires land evaluation [3]. Land suitability evaluation is valuable for identifying critical limits of crop yield [25, 26], devising sustainable land management options for improving productivity of land [26, 27], and mitigating food security problems [21]. Its significance needs to be more urgent to the sub-Sahara nations of Africa such as Ethiopia where food insecurity problem is still challenging [21].

In Ethiopia, land is often exploited traditionally without analyzing the costs incurred and benefits (profits) earned [7] between competing uses or purposes [9]. This is a critical problem where land is utilized by smallholders, who constitute the greatest share of the agrarian society in the country [6, 7]. In such cases, land may not be exploited for the appropriate use (purpose) from which the optimum economic return is earned [4, 17]. Farmers' experience in Sinana area, southeastern Ethiopia, is not different from the smallholder farmers in other parts of the country. Most farmers of the area practice farming traditionally without scientific knowledge about the land exploited. Thus, it is essential to evaluate land suitability of SRS for specific land use types in terms of physical requirements [3, 9].

The government of Ethiopia has strong ambition to increase production of cereals particularly wheat to ensure national food security as its national strategy and realize its vision of becoming a wheat export nation. Sinana is among the surplus wheat producing area in the area. The innovative element of this study is the use of ten land characteristics for the two major cereals (wheat and barley), which is useful for making decisions related to sustainable agricultural development. Therefore, the study provided evidence-based data for sustained and quality agricultural development in area which in turn accelerates economic progress. Result of the study will point to policy makers and planners at local, regional, and national levels about the key land use threats and its management options. This study was targeted to (a) evaluate the level of suitability of SRS for wheat and barley farming using selected soil and topographic and climate parameters, (b) compare the cumulative level of land suitability of the area for both land uses, and (c) assess the critical threats of land suitability for wheat and barley farming in the study site.

2. Materials and Methods

2.1. Description of the Study Area. The “study area,” Sinana Research Site (SRS), is located within 07°06′12″–07°07′29″N and 40°12′40″–40°13′52″E. It is found in Sinana district, Bale Zone in Oromia Regional State, southeastern Ethiopia. SRS has a total area of 887 ha (including the uncultivated land), and its cultivated land is 207.87 ha (Figure 1).

The altitude of Sinana district is 1843 m–3250 m above sea level. It has *Woina-Dega* (sub-tropical) to *Dega* (temperate) agro-climate. But the “study area” (SRS) used for evaluation is 1843 m–2300 m high above sea level, which reflects a *Woina-Dega* (sub-tropical) agro-climate [28]. The majority of the ‘study area’ has a slope gradient below 3° (5%), and its gradient is 100% less than 6° (10%). The area receives mean total annual rainfall of 856 mm with bimodal pattern, where rainfall peaks in April to May and August to October. Mean annual temperature (1990–2018) of the “study site” was 15.4°C, and it ranges from 14.8°C (in 1993) to 15.8°C (in 1998). Major soils of the area are Phaeozems (71%), Cambisols (25.2%), and Vertisols (3.8%) [28, 29].

Both livestock and crop production are practiced in Sinana area. It is one of the major cereal growing regions of Ethiopia. The area is highly known in barley and wheat production [30]. The Central Statistical Authority (CSA) reported that population of Sinana district was about 165,768; of whom, about 86,250 (52%) were males and 79,518 (48%) were females [31].

2.2. Data and Methods

2.2.1. Assumptions and Selection of Land Characteristics Used for Evaluation. This land suitability evaluation was made for wheat and barley production in SRS. Wheat and barley are the two dominant crops in the district in terms of production, cultivated area, and consumption [32]. The land in the area is utilized by smallholder farmers under the traditional farming system with low farm inputs; land is used for wheat production which is harvested in about 150 days (June to October) and barley cultivation which is harvested in 120–150 days (June to September/October) [1, 2, 9].

The study is a physical suitability evaluation based on soil, topography, and climate land characteristics [3] such as (i) chemical soil properties such as nitrogen (N), phosphorous (P), potassium (K), cation exchange capacity (CEC), and soil pH, (ii) physical soil properties (depth and drainage), (iii) topographic (slope gradient), and (iv) climate (growing period temperature and rainfall) parameters.

Selection of the land characteristics used for evaluation was determined by data availability, level of significance of the attributes in influencing the land suitability of wheat and barley, and the occurrence of critical values of the attributes in the study area [3, 9]. Temperature was used to represent adequacy of energy for the land uses considered in the evaluation; rainfall represents the land quality ‘adequacy of moisture/water” requirement for wheat and barley farming [3]. Previous studies showed that climate variability, land degradation, and declining soil fertility contributed to deterioration of the livelihood of smallholder farmers in

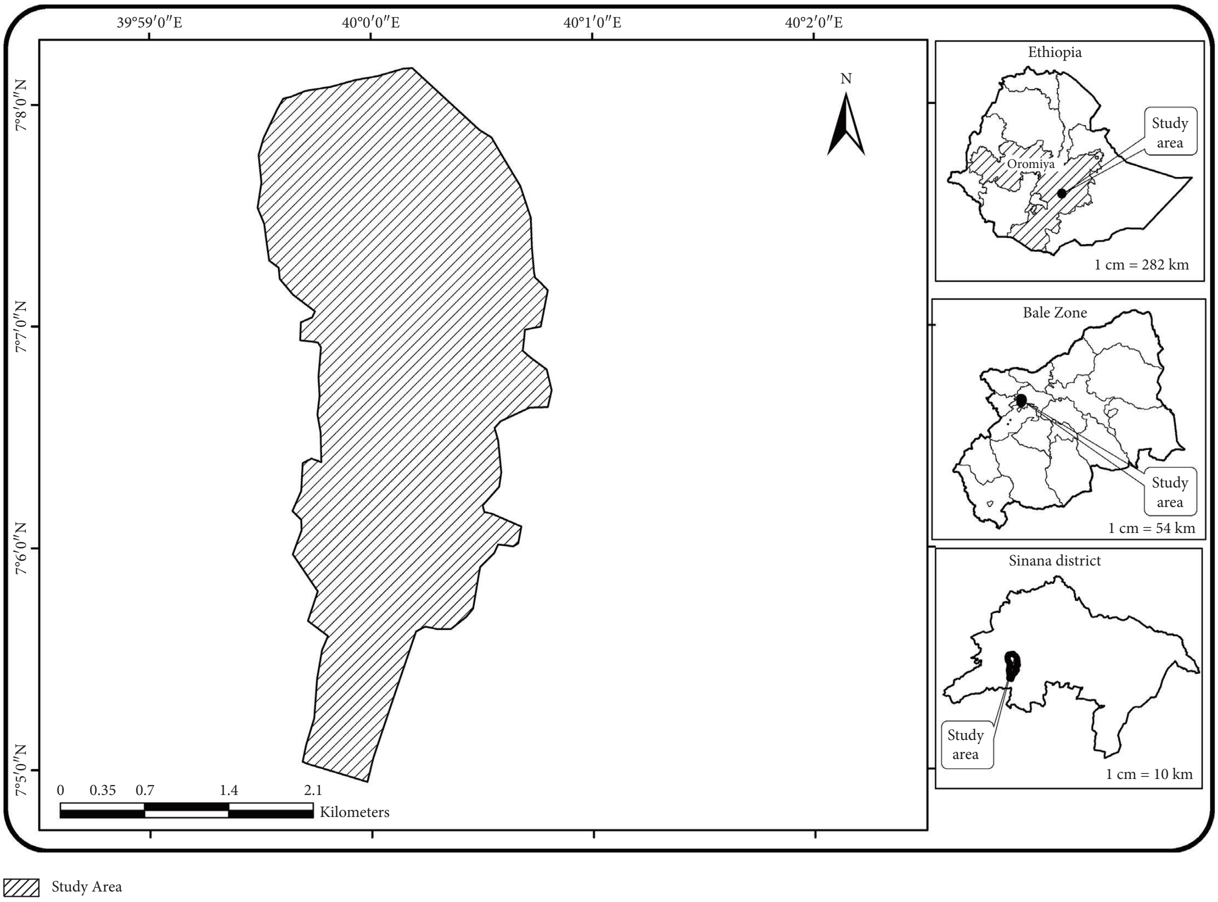

Figure 1: Location of the study area.

Ethiopia [22]. The ten land characteristics used belongs to these parameters and are selected with this justification. Moreover, similar land characteristics have been used in different studies [33–39].

2.2.2. Data Sources and Method of Acquisition. Soil and topographic data required for this study were obtained largely through land and soil survey, which was conducted in SRS, southeastern Ethiopia [40]. Data about total N (Kjeldahl method), available P (Olsen's 0.5 mole sodium bicarbonate, pH 8.5), exchangeable K (1 mole ammonium acetate, pH 7), CEC (1 mole ammonium acetate, pH 7), soil pH (potentiometer, using H_2O), and texture (the modified Bouyoucos hydrometer method) were determined using the laboratory test of 15 *composite soil samples* taken from 33 sample sites [40]. Soil depth was measured using auger, and slope gradient was generated from elevation data traced using GPS [40]. Climate (growing period temperature and rainfall) data were obtained from national meteorological agency of Ethiopia for the period 1990–2018.

2.2.3. Methods of Analysis. Criteria were set for each of the ten land characteristics with respect to the requirements of wheat and barley (Tables 1 and 2) using crop-environment manuals [1, 41]. Suitability levels of sample sites (areas), with reference to each parameter, were rated as S1 (highly suitable), S2 (moderately suitable), S3 (marginally suitable), and/or N (not suitable) (Tables 1 and 2 and Figure 2) depending on the management and input cost required [3]. The "land characteristics" were matched against the "land use requirements" of wheat and barley farming to specify the level of land suitability for both crops separately. The decision-making process for determining the overall suitability of the area using the "maximum limiting factor" technique is a valuable approach since it is an important tool for land suitability evaluation around the world [43]. Specifically, the "maximum limiting factor" method is used in Ethiopia by FAO [1–3], Ayalew and Silassie [17]; Girmay et al. [5, 39], Moshago et al. [44] to evaluate land suitability for agricultural crops. The method was used to rate the level of cumulative suitability class of each sample area-unit for each land use with reference to all the attributes. Interpretations and discussions were made about the suitability of land for both land uses.

3. Results and Discussion

3.1. Suitability of the Sinana Research Site (SRS) for Wheat and Barley Farming Using Ten Land Characteristics. N content of soils influences productivity of crops by affecting chlorophyll development and photosynthetic process by crop plants. Soils of SRS are characterized by deficient N supply (Table 3).

Table 1: Evaluation criteria for the land suitability analysis for wheat farming.

No.	Land characteristics	Suitability class			
		S1	S2	S3	*N*
1	Nitrogen _N (%)	>0.50	0.10–0.50	0.02–0.10	<0.02
2	Phosphorous _P (kg P ha^{-1})	>15	8–15	3–8	<3
3	Potassium _K (meq/100 g)	>0.50	0.20–0.50	0.02–0.20	<0.02
4	CEC (me/100 g)	>40	25–40	15–25	<15
5	Soil pH	6.00–8.00	5.50–6.00 and 8.00–8.20	5.00–5.50 and 8.20–8.50	<5.00 and >8.5
06	Slope gradient (%)	<5	5–10	10–30	>30
7	Drainage/texture	Well-drained	Moderately D	Poorly drained	Not drained
8	Soil depth (cm)	100–150	50–100	30–50	<30
9	Mean of GP temp (0C)	14–17	12–14 and 17–20	10–12 and 20–24	<10 and >24
10	Total GP rainfall (cm)	60–75	45–60 and 75–90	30–45 and 90–105	<30 and >105

Source: own design based on FAO [1]; Landon [41]; NMA [42]. *Note.* GP = growing period.

Table 2: Evaluation criteria for the land suitability analysis for barley farming.

No	Land characteristics	Suitability class			
		S1	S2	S3	*N*
1	Nitrogen _N (%)	>0.40	0.20–0.40	0.10–0.20	<0.10
2	Phosphorous _P (kg P ha^{-1})	>15	8–15	3–8	<3
3	Potassium _K (meq/100 g)	>0.20	0.10–0.20	0.08–0.10	<0.08
4	CEC (me/100 g)	>40	25–40	15–25	<15
5	Soil pH	6.50–7.80	6.00–6.50 and 7.80–8.00	5.50–6.0 0 and 8.00-8.50	<5.50 and > 8.50
6	Slope gradient (%)	<5	5–10	10–30	>30
7	Drainage/texture	Well drained	Moderately drained	Poorly drained	Not drained
8	Soil depth (cm)	>100	50–100	30–50	<30
9	Mean of GP temp (0C)	14–17	12–14 and 17–20	10–12 and 20–24	<10 and >24
10	Total GP rainfall (cm)	60–75	45–60 and 75–90	30–45 and 90–105	<30 and >105

Source: own design based on FAO [1]; Landon [41]; NMA [42]. *Note.* GP = growing period.

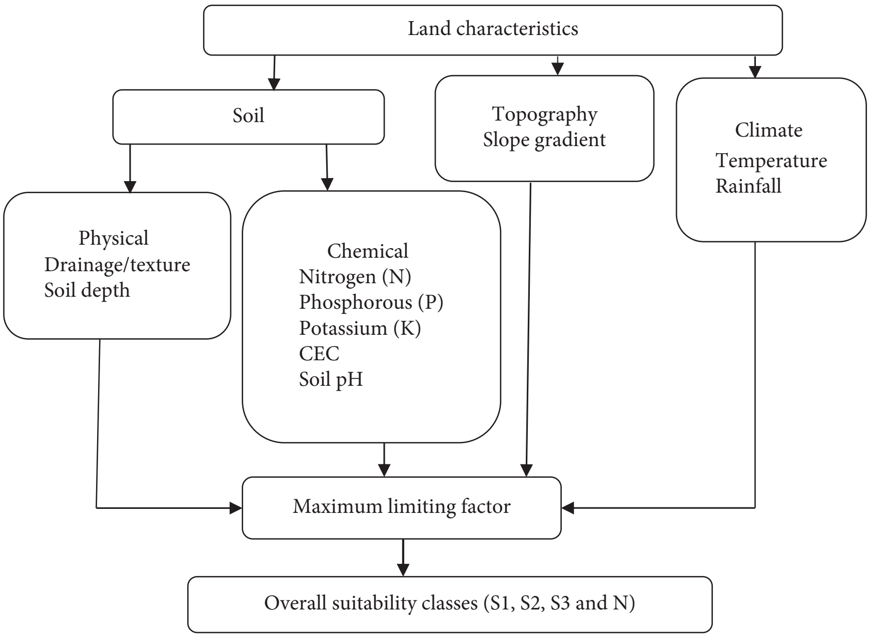

Figure 2: The land evaluation model.

As it is illustrated in Table 3, about 96.2% of the SRS was "marginally suitable" (S3) and only insignificant proportion (3.8%) of the area was "moderately suitable" (S2) for both wheat and barley production. There is no difference in the level of suitability of land between wheat and barley in terms of the requirement of nitrogen in the study area.

Table 3: Land suitability for wheat and barley farming using nitrogen (N) requirement.

Crops	Land characteristics	Requirement (LUR)	Samples	Area (ha)	Area (%)	Class
Wheat	Total N (%)	>0.50	0	0.00	0.00	S1
		0.10–0.50	1	7.96	3.80	S2
		0.02–0.10	14	199.91	96.20	S3
		<0.02	0	0.00	0.00	*N*
Barley	Total N (%)	>0.40	0	0.00	0.00	S1
		0.20–0.40	1	7.96	3.80	S2
		0.10–0.20	14	199.91	96.20	S3
		<0.10	0	0.00	0.00	*N*

Note. LUR = requirement.

Production of wheat and barley is also constrained by the status of available phosphorus even if the degree of limitation is not as severe as that of nitrogen. Based on available phosphorous supply, about 42.1% and 46.5% of the area was "moderately suitable" (S2) and 'marginal suitability" (S3) for wheat farming, respectively. Limited share (11.4%) of the area was "not suitable" (N) for the same crop (wheat).

Similarly, significant share of the study area was "moderately" (42.1%) and "marginally" (46.5%) suitable for barley production (Table 4). No part of the area was "highly suitable" for both wheat and barley farming. In other words, SRS showed no suitability difference between wheat and barley farming in terms of available P; but deficiency of this nutrient was equally a critical threat on the suitability of land for both crops. Removal of crop residue for livestock fodder, limited use of green manure, and lack of crop rotation with legumes could be causes for deficient P and N in the soil; this is because, over 90% of N and P supply is generally derived from decomposition of organic matter in the soil [18, 45].

The level of exchangeable K exceeds 0.5 me/100 g soil throughout the study area. Thus, in terms of exchangeable K, 100% of the area was "highly suitable" (S1) for optimum production of wheat and barley (Table 5). The status of exchangeable K is rated "high" if it exceeds 0.5 me/100 g soil, and hence, application of chemical fertilizer is often unnecessary [41].

Cation exchange capacity (CEC) is a measure of the amount of negatively charged particles on the exchange (clay-humus) surface in the soil; in other words, it indicates the nutrient storing and exchanging capacity of the soil [45]. In terms of the requirement of CEC, 100% of the study area was "highly suitable" (S1) for the cultivation of wheat. Similarly, the whole SRS was "highly suitable" for sustainable barley production (Table 6). The implication is that there was little variation in land suitability between wheat and barley in terms of the requirement for CEC.

Soils of the study area have high nutrient storage capacity where values of CEC of all sample sites were measured within 37.0–58.2 (cmol(+)kg^{-1} soil). Results of the soil pH-based suitability evaluation of SRS for wheat and barley farming are organized in Table 7.

The level of land suitability for wheat production was rated "highly" (S1), "moderately" (S2), "marginally" (S3), and "not" (N) suitable in about 21.7%, 19.4%, 37.7%, and 21.1% of the area, respectively, in terms of the requirement of soil reaction (pH) (Table 7). Soil reaction (pH) is a vital indicator of suitability and productivity of land as it controls the form of nutrient availability or unavailability and the base saturation (Ca^{++}, Mg^{++}, K^{+}, and Na^{+}) in the soil and as it also reflects the toxicity of soil [9, 23, 45].

Based on soil pH requirement, the area that is "highly suitable" (31.9%) for barley production was about 10.1% larger than the area under the "highly suitable" (21.7%) category for wheat farming. The area which was "marginally suitable" (42.6%) for barley was slightly larger than the same suitability class (37.7%) for wheat production (Table 7). That is, there was limited overall suitability difference between wheat and barley production based on soil reaction (pH) as a criterion, except for the variation in S1 and S2 suitability levels of the crops.

Slope gradient is the other key parameter used for the suitability evaluation (Table 8). While about 86.7% of the district was "highly suitable" for wheat and barley farming, only 13.3% of the area was "moderately suitable" for the production of both crops in the requirement of slope gradient (Table 8). In fact, topography, being the function of slope length and gradient, is not a critical constraint on both wheat and barley production in the area. Here, only 13.3% of the area, where the gradient is 3°–6°, was rated at "moderate suitability" level implies that the adverse effect of topography or slope gradient on the optimum yield of wheat and barley is limited; that is, slope gradient influences suitability of land by increasing or decreasing incidence of erosion hazard, stability of soil, and impacting soil depth [9].

Soil drainage is the other attribute used to measure the level of suitability of land for wheat and barley farming. Soil drainage can be measured by soil texture composition (Table 9). About 96.2% was "highly suitable" for both wheat and barley production by status of soil drainage; that is, the largest share of the area was well drained to ensure adequate air and moisture circulation for roots of crops. Only a small portion (3.8%) of the area was "moderately suitable" for both land use types (that is, wheat and barley). There was no difference in the level of land suitability between wheat and barley based on drainage/texture requirement (Table 9).

Soil depth is a vital attribute used to measure the level of suitability of land for a certain production in a locality. Slightly less than three-fourth (71.9%) of the SRS was "highly suitable" (S1) for the optimum production of wheat and barley since the soil depth exceeds one meter (>100 cm). The remaining 28.1% of the area was "moderately suitable" (S2)

Table 4: Land suitability for wheat and barley farming using phosphorous (P) requirement.

Crop	Land characteristic	Requirement (LUR)	Samples	Area (ha)	Area (%)	Class
Wheat	Available P (kg P ha^{-1})	>15	0	0.00	0.00	S1
		8–15	4	87.55	42.10	S2
		3–8	8	96.69	46.50	S3
		<3	3	23.63	11.40	*N*
Barley	Available P (kg P ha^{-1})	>15	0	0.00	0.00	S1
		8–15	4	87.55	42.10	S2
		3–8	8	96.69	46.50	S3
		<3	3	23.63	11.40	*N*

Note. LUR = requirement.

Table 5: Land suitability for wheat and barley farming using potassium (K) requirement.

Crops	Land characteristics	Requirements (LUR)	Samples	Area (ha)	Area (%)	Class
Wheat	Exchangeable K (me/100 g)	>0.50	15	207.90	100.00	S1
		0.20–0.50	0	0.00	0.00	S2
		0.02–0.10	0	0.00	0.00	S3
		<0.02	0	0.00	0.00	*N*
Barley	Exchangeable K (me/100 g)	>0.20	15	207.87	100.00	S1
		0.10–0.20	0	0.00	0.00	S2
		0.08–0.10	0	0.00	0.00	S3
		<0.08	0	0.00	0.00	*N*

Note. LUR = requirement.

Table 6: Land suitability for wheat and barley farming using the requirement of CEC.

Crops	Land characteristics	Requirements (LUR)	Samples	Area (ha)	Area (%)	Class
Wheat	CEC (me/100 g)	>40	14	207.87	100.00	S1
		25–40	1	0.00	0.00	S2
		15–25	0	0.00	0.00	S3
		<15	0	0.00	0.00	*N*
Barley	CEC (me/100 g)	>40	14	207.87	100.00	S1
		25–40	1	0.00	0.00	S2
		15–25	0	0.00	0.00	S3
		<15	0	0.00	0.00	*N*

Note. LUR = requirement.

Table 7: Land suitability for wheat and barley farming using soil pH requirement.

Crop	Soil pH description	Requirement (LUR)	Sample	Area (ha)	Area (%)	Class
Wheat	Slightly acidic, neutral and SA	6.0–8.00	2	45.21	21.80	S1
	Moderately alkaline	8.00–8.20	3	40.41	19.40	S2
	Strongly alkaline	8.20–8.50	5	78.32	37.70	S3
	Very strongly alkaline	>8.50	5	43.93	21.10	*N*
Barley	Neutral and slightly alkaline	6.50–7.80	6	66.26	31.90	S1
	Slightly acidic	6.0–6.50 and 7.8–8.00	1	14.68	7.10	S2
	Slightly acidic and alkaline	5.50–6.00 and 8.0–8.50	7	88.63	42.60	S3
	Strongly acidic and alkaline	<5.50 and >8.50	1	38.30	18.40	*N*

Note. SA = slightly alkaline; LUR = requirement.

for both land uses upon the requirement of soil depth (that is, where soil depth was 50–100 cm) (Table 10). The soil depth should exceed 100 cm for the optimum productivity of land for wheat and barley farming [1, 41]. This is because deep soils (depth of 100–150 cm or more) provide adequate space for good root growth, nutrients supply, and moisture retention for the cereals.

Temperature is an indicator of energy sufficiency for the growth of crops [3]. The result of the suitability assessment revealed that 100% (207.87 ha) of the arable land of the study site is "highly suitable" for the optimum production of both wheat and barley (Table 11). The mean temperature during the growing period (June–October) of wheat and barley cultivation in SRS (which was averaged for years 1990–2018)

Table 8: Land suitability for wheat and barley using the requirement of slope gradient.

Crops	Land characteristics	Requirements (LUR)	Samples	Area (ha)	Area (%)	Class
Wheat	Slope gradient (%)	0–5	11	180.18	86.70	S1
		5–10	4	27.69	13.30	S2
		10–30	0	0.00	0.00	S3
		>30	0	0.00	0.00	*N*
Barley	Slope gradient (%)	0–5	11	180.18	86.70	S1
		5–10	4	27.69	13.30	S2
		10–30	0	0.00	0.00	S3
		>30	0	0.00	0.00	*N*

Note. LUR = requirement.

Table 9: Land suitability for wheat and barley farming using drainage requirement.

Crops	Land characteristics	Requirements (LUR)	Samples	Area (ha)	Area (%)	Class
Wheat	Drainage (texture)	Well-drained	14	199.91	96.20	S1
		Moderately	1	7.96	3.80	S2
		Poorly drained	0	0.00	0.00	S3
		Not drained	0	0.00	0.00	*N*
Barley	Drainage (texture)	Well-drained	14	199.91	96.20	S1
		Moderately	1	7.96	3.80	S2
		Poorly drained	0	0.00	0.00	S3
		Not drained	0	0.00	0.00	*N*

Note. LUR = requirement.

Table 10: Land suitability for wheat and barley farming using soil depth requirement.

Crops	Land characteristics	Requirements (LUR)	Samples	Area (ha)	Area (%)	Class
Wheat	Soil depth (cm)	>100	11	149.51	71.90	S1
		50–100	4	58.36	28.10	S2
		30–50	0	0.00	0.00	S3
		<30	0	0.00	0.00	*N*
Barley	Soil depth (cm)	>100	11	149.51	71.90	S1
		50–100	4	58.36	28.10	S2
		30–50	0	0.00	0.00	S3
		<30	0	0.00	0.00	*N*

Note. LUR = requirement.

Table 11: Temperature requirement-based land suitability for wheat and barley farm.

Crops	Land characteristics	Requirements (LUR)	Samples	Area (ha)	Area (%)	Class
Wheat	Mean GP temperature (°C)	14–17	15	207.87	100.00	S1
		12–14 and 17–20	0	0.00	0.00	S2
		10–12 and 20–24	0	0.00	0.00	S3
		<10 and >24	0	0.00	0.00	*N*
Barley	Mean GP temperature (°C)	14–17	15	207.87	100.00	S1
		12–14 and 17–20	0	0.00	0.00	S2
		10–12 and 20–24	0	0.00	0.00	S3
		<10 and >24	0	0.00	0.00	*N*

Note. GP = growing period; LUR = requirement.

was 15.3°C, and it ranges 14.3°C–16.8°C within the three decades. Thus, the growing period temperature of the study area is ideal for the production of both cereal crops.

The result of the wheat and barley suitability evaluation of the area in terms of the mean total rainfall during the growing period of the crops is shown in Table 12. The result of the rainfall requirement-based evaluation of SRS was different from that of the temperature-based suitability evaluation; that is, the whole arable land (100%) was only moderately suitable (S2) for the optimum production of both wheat and barley.

Normally, wheat grows well in localities where mean total rainfall during its growing period is about 600 mm (60 cm) [42]. Similarly, barley provides optimum yield

Table 12: Growing period rainfall-based land suitability for wheat and barley farming.

Crops	Land characteristics	Requirements (LUR)	Samples	Area (ha)	Area (%)	Class
Wheat	Mean TGP rainfall (cm)	60–75	0	0.00	0.00	S1
		45–60 and 75–90	15	207.87	100.00	S2
		30–45 and 90–105	0	0.00	0.00	S3
		<30 and >105	0	0.00	0.00	*N*
Barley	Mean TGP rainfall (cm)	60–75	0	0.00	0.00	S1
		45–60 and 75–90	15	207.87	100.00	S2
		30–45 and 90–105	0	0.00	0.00	S3
		<30 and >105	0	0.00	0.00	*N*

Note. TGP = total growing period; LUR°=°requirement.

where mean total rainfall during its growing period is 600–700 mm (65–70 cm), which is well distributed within the growing season [42]. Averaged for years 1990–2018, mean total rainfall of SRS during the growing period of wheat (June–October) and barley (June–September/October) was 480.7 mm (403.0–628.1 mm). Barely tolerates wide variation in rainfall amount than wheat. Generally, there is no suitability difference between wheat and barley farming based on the requirements of energy (temperature) and moisture (rainfall) adequacy.

3.2. Overall Land Suitability for Wheat and Barley Production. Results of the overall suitability of SRS for wheat and barley farming are reported based on the ten (10) land characteristics used for evaluation. Land, in Sinana area, is less suitable for the production of wheat. Slightly less than three-fifth (57.6% or 119.73 ha) of the study site was only "marginally suitable" (S3) for wheat farming (Table 13), that is, where N deficiency, low available P, and alkalinity problems of soils were the maximum limiting factors in 53.7%, 21.2%, and 35.8% of the area with the "marginal" level of suitability for wheat production, respectively (Table 13).

On the other hand, about 31% (64.51 ha) of the total area of SRS was "not suitable" (N) for wheat cultivation primarily due to strongly alkaline soils (high pH). Similarly, about 11.4% (23.63 ha) of the district was "not suitable" (N) for the sustainable production of wheat because of critical deficiency of available P in the soil (Table 13). Here, one of the basic reasons for the deficient available P in the area could be the strongly alkaline soils; for instance, soil pH was 7.3–9.1, 7.4–8.7, and 6.7–8.6 in about 2.1%, 3.7%, and 5.8% of the study area, respectively, and the ideal soil pH requirement for the optimum production (high suitability) of wheat is 6.0–8.0 [41]. Thus, available P becomes deficient in the alkaline environment (or strongly alkaline soils) since it is often fixed by calcium and magnesium carbonates where soil pH exceeds 8.0–8.5 [41, 45]. Wheat farming in Sinana area, to some extent, is also threatened by rainfall scarcity even if it (rainfall) is not a "maximum limiting factor" on suitability of land for this crop.

Conditions of attributes such as drainage, soil depth, slope gradient, CEC, exchangeable K, and temperature were ideal for the optimum production of wheat in large part of Sinana area. Soils in 96.2% of the study area were well drained excepting the very sticky and moderately drained Eutric Vertisols, covering only 3.8% area (Table 13). The

Table 13: Result of the overall land suitability evaluation for wheat farming.

No.	Sample size	Overall suitability level	Area (ha)	Area (%)
1	1	S3n	37.25	17.90
2	3	Np	23.63	11.40
3	7	NpH	64.51	31.00
4	2	S3pnpH	36.22	17.40
5	1	S3npH	38.30	18.40
6	1	S3p	7.96	3.80
Total	15		207.87	100.00

Note. S3n = marginally suitable because of nitrogen. Np = not suitable because of phosphorous. NpH = not suitable because of soil pH. S3pnpH = marginally suitable because of phosphorous, nitrogen, and soil pH. S3npH = marginally suitable because of nitrogen and soil pH. S3p = marginally suitable because of phosphorous.

potential effect of runoff erosion on the suitability of land for wheat farming is not a serious problem in large part of the study area where the maximum slope gradient was 6° (10%) and the area of the study site with slope gradient 3–6° (5–10%) was only 13.3%.

The result of the overall suitability evaluation for barley farming is also summarized in Table 14. Large part of the arable land of SRS was also "marginally suitable" for barley production. That is, about 70.1% of the study area was "marginally suitable" (S3) for barley production. Here, attributes like N deficiency in 66.4%, deficiency of available P in 46.5%, and moderately alkaline soils in 42.7% were the maximum limiting factors or constraints for the greatest share of the area to have been rated "marginally suitable" (S3) for barley production (Table 14). About 18.4% of the arable land of SRS was "not suitable" (N) for production of barley due to limitation in soil reaction (pH), that is, where the same share (%) of the area was featured by strongly alkaline soil that restrict suitability of land for the land use type considered. Similarly, about 11.4% of the study area was also "not suitable" (N) for barley faming because of critical deficiency of available P in the soil (Table 14). Limited recycling of crop residue is among the causes of deficient N and P supply. In this regard, a study showed that depletion of organic matter is usually due to use of crop residue for animal fodder, fuel, building home, etc., purposes in most parts/areas of Ethiopia [18].

The well-drained soils (that is, clay and clay-loam texture in 96.2% area), the gentle-to-plain landscape with slope gradient of less than and equal to 3° (in 86.7% area), and less

Table 14: Result of the overall land suitability evaluation for barley farming.

No.	Sample size	Overall suitability level	Area (ha)	Area (%)
1	1	S3n	37.25	17.90
2	3	Np	23.63	11.40
3	5	S3pnpH	76.63	36.90
4	1	NpH	38.30	18.40
5	2	S3npH	12.00	5.80
6	2	S3pn	12.10	5.80
7	1	S3p	7.96	3.80
Total	15		207.87	100.00

Note. S3n = marginally suitable because of nitrogen. Np = not suitable because of phosphorous. S3pnpH = marginally suitable because of phosphorous, nitrogen and soil pH. NpH = not suitable because of soil pH. S3npH = marginally suitable because of nitrogen and soil pH. S3pn = marginally suitable because of phosphorous and nitrogen. S3p = marginally suitable because of phosphorous.

than and equal to 6° (in 100% area), the deep soils with 100–150$^+$ cm (72% area), the rich exchangeable K with 0.96–3.44 me/100 g soil (100% area), the high CEC with a value of 37.0–58.2 (cmol(+)kg^{-1} soil) (97.9% area), and temperature (100%) have a limited effect on the land suitability of barley. Rainfall scarcity threatens barley farming to some level although it is not a "maximum limiting factor" on the land suitability of the cereal in the area. Everest et al. [46] underlined that future climate adaptation strategies should take into account results of land suitability assessment.

The greatest share of SRS was only "marginally suitable" for wheat (57.6%) and barley (70.2%) farming, and this is slightly similar to the result of a study made in Midlands of Tigray (northern Ethiopia) where the evaluation was made for barley, maize, etc., crops [26]. But the result of our study was significantly different from the findings of another study made in Jello Catchment (eastern Ethiopia) where 48–67% of the area was "not suitable" for the crops (maize, wheat, and sorghum) evaluated [4].

While slightly less than three-fifth of SRS was "marginally suitable" (S3) for wheat farming, over two-third of the area was with the same level of suitability (S3) for barley. Significant share of the area was "not suitable" for wheat (42.4%) and barley (29.8%) cultivation. Soil pH (alkalinity) and deficiency of N and available P were critical threats on the suitability of land for the production of the cereal crops accounted for evaluation. Even if rainfall scarcity is not a "maximum limiting factor" on the suitability of SRS for wheat and barley farming, it is proved to threaten yield of the cereal crops to some level. The slightly-to-strongly alkaline soils should have led to deficiency of available P and total N supply in soils of the study site as most nutrients, including P and N, exist in an adequate supply (or available forms) where soil pH is around neutrality (at pH 7). Frequent cultivation of similar crops/cereals on the same farmland in consecutive seasons, limited green manuring, retarded recycling of organic matter, and use of crop residue as fodder for livestock (or use of croplands for grazing immediately after harvest) could also be causes for low N and available P supply. Had there been adequate nutrient recycling from crop residue, soils of the Sinana site could have been rich in total N and available P supply since up to 98% of the total N and over 90% of the available P in the soil can be derived from organic matter.

Here are the possible solutions to mitigate N and P deficiency and alkalinity problems of soils (1) chemical fertilizers: this has to be the ultimate solution where there are no other ways of increasing N and P supply in the soil; (2) organic fertilizer (manure and compost): this provides multiple benefits: first, it enhances the level of N and P in the soil as the greatest proportion of these nutrients can be gained from organic substances; second, decomposition of fresh organic matter releases "humic acid" (citrus, tartar, acetate, etc.), which reduces the soil pH since it has a neutralizing effect on alkaline soils; and third, organic matter improves nutrient storage and moisture retention capacity of soils and thus increased moisture retention implies rising concentration of H+, which in turn implies increasing acidity and reducing alkalinity. (3) Rotating leguminous crops with wheat and barley improves the soil nutrient status as legumes have root nodules that can fix nitrate (NO3) and ammonia (NH3) in the soil. (4) Green manuring: this could be integrated with option number (2) above; anyway, green manuring is useful to improve nutrient supply and reduce alkalinity of soils. Hence, farms under "marginally suitable" (S3) and "not suitable" (N) category can probably be improved to "moderate" (S2) and/or "high" (S1) level of suitability.

A study by Debesa et al. [35] on physical land suitability analysis for major cereal crops in Dabo Hana district, South-West Ethiopia, reported that altitude, temperature, and rainfall are the dominant factors that influence the suitability of agricultural land for the major cereals grown in their study area. Fekadu and Negese [47] on the other hand reported that soil pH, AWC, TN, and available P were identified as the principal limiting factors for wheat and barley crops in Yikalosub watershed, Ethiopia. Another study by Debalke et al. [48] identified soil organic matter, temperature, length of growing season, rainfall, soil depth, and pH to be the primary limiting elements preventing the cultivation of wheat, barley, and teff in the Arsi zone of Ethiopia. Decisions for effective agricultural planning can be benefited more from crop-based land suitability studies for micro land areas, instead of land assessments of macro land areas [46]. Sustainable agricultural production has become the key responsibility of agricultural development planners as land resources of the world have reached ultimate limits [49]. Therefore, crop-based land suitability assessment is useful for growing crops where they are best suitable [46], which demands a comprehensive knowledge of land characteristics [30]. This in turn is helpful to achieve food security for the growing population [46]. In general, the above studies indicated that similar land suitability assessment should be conducted for different crops and other areas as well. The study effectively evaluates the physical land characteristics and their suitability for wheat and barley production, with the aim of improving local food security in certain regions. The findings provide

practical recommendations for improving agricultural practices. Overall, this research contributes to the knowledge on sustainable agriculture practices and offers valuable insights for policymakers and farmers in the region.

The innovative element of this study is the proposed mitigation strategies to address the identified constraints. Furthermore, the study emphasizes the potential for improving the farms categorized as "marginally" and "not" suitable to achieve a moderate (S2) or high (S1) level of suitability for the studied crops. By implementing the suggested strategies, such as optimizing fertilizer usage and adopting appropriate crop rotation practices, it is anticipated that the yield and food security status of households in the SRS can be positively impacted.

4. Conclusions

This study has evaluated suitability of SRS (southeastern Ethiopia) for sustainable wheat and barley production to improve local food security. The suitability evaluation has enabled to identify the key constraints of land for the optimum yield of wheat and barley in Sinana area. Fortunately, the land use problems of SRS are those about which management options are not impossible; there are options that could be applied by farmers for surmounting land use threats of the cereal crops studied and hence to improve yield of the crops. Hence, farms under "marginally suitable" (S3) and "not suitable" (N) category can probably be improved to the "moderate" (S2) and/or "high" (S1) level of suitability. This, in turn, would contribute to increment in yield of the cereal crops and improvement in food security status of people in the study area. While this study provides a comprehensive evaluation of land suitability for wheat and barley production in southeastern Ethiopia, it does not provide an explanation on the evolution of yields for the two crops over the period of analysis. Further studies are needed to map the specific land characteristics to ease decisions in the agricultural planning.

Data Availability

The data used to support the findings of this study are included within the article.

Ethical Approval

This article does not contain any studies with human participants or animals performed by the author.

Conflicts of Interest

The authors declare that they have no conflicts of interest.

Acknowledgments

The author would like to thank Abren Gelaw (PhD) for his support in analyzing soil data. The paper has benefited from useful comments and suggestions by the anonymous referees to whom the author is very grateful.

References

[1] FAO (Food and Agriculture Organization), *A Guideline of Land Evaluation for Rain-Fed Agriculture: Crop-Environment Requirements in Ethiopia*, vol. 2, Food and Agriculture Organization, Addis Ababa, Ethiopia, 1983.

[2] FAO (Food and Agriculture Organization), *A Framework for Land Evaluation*, Food and Agriculture Organization, Rome, Italy, 1984.

[3] FAO (Food and Agriculture Organization), *Guidelines for Land Use Planning*, Food and Agricultural Organization, Rome, Italy, 1996.

[4] R. Girma, A. Moges, and S. Quraishi, "GIS based physical land suitability evaluation for crop production in Eastern Ethiopia: a case study in Jello Watershed," *Agrotechnology*, vol. 5, no. 1, pp. 1–7, 2015.

[5] G. Girmay, W. Sebnie, and Y. Reda, "Land capability classification and suitability assessment for selected crops in Gateno watershed, Ethiopia," *Cogent Food and Agriculture*, vol. 4, no. 1, Article ID 1532863, 2018.

[6] A. Kahsay, M. Haile, G. Gebresamuel, and M. Mohammed, "Land suitability analysis for sorghum crop production in northern semi-arid Ethiopia: application of GIS-based fuzzy AHP approach," *Cogent Food and Agriculture*, vol. 4, no. 1, Article ID 1507184, 2018.

[7] S. G. Yalew, A. van Griensven, M. L. Mul, and P. van der Zaag, "Land suitability analysis for agriculture in the Abbay basin using remote sensing, GIS and AHP techniques," *Modeling Earth Systems and Environment*, vol. 2, no. 2, p. 101, 2016.

[8] A. Sarkar, A. Ghosh, and P. Banik, "Multi-criteria land evaluation for suitability analysis of wheat: a case study of a watershed in eastern plateau region, India," *Geo-spatial Information Science*, vol. 17, no. 2, pp. 119–128, 2014.

[9] Fao (Food and Agriculture Organization), *Land Evaluation: Towards a Revised Framework*, Food and Agriculture Organization, Rome, Italy, 2007.

[10] W. Bewket, "Land cover dynamics since the 1950s in Chemoga watershed, Blue Nile basin, Ethiopia," *Mountain Research and Development*, vol. 22, no. 3, pp. 263–269, 2002.

[11] P. Dubale, "Soil and water resources and degradation factors affecting productivity in Ethiopian highland agro-ecosystems," *Northeast African Studies*, vol. 8, no. 1, pp. 27–51, 2001.

[12] Fao (Food and Agriculture Organization), *Analysis of Price Incentives for Teff in Ethiopia; Technical Notes Series; MAFAP*, B. Assefa, M. Demeke, and B. Lanos, Eds., Food and Agriculture Organization, Rome, Italy, 2015.

[13] H. Hurni, K. Tato, and G. Zeleke, "The implications of changes in population, land use, and land management for surface runoff in the upper Nile basin area of Ethiopia," *Mountain Research and Development*, vol. 25, no. 2, pp. 147–154, 2005.

[14] M. Lemenih, E. Karltun, and M. Olsson, "Assessing soil chemical and physical property responses to deforestation and subsequent cultivation in smallholders farming system in Ethiopia," *Agriculture, Ecosystems and Environment*, vol. 105, no. 1-2, pp. 373–386, 2005.

[15] B. Tegene, "Land-cover/land-use changes in the derekolli catchment of the South welo zone of amhara region, Ethiopia," *Eastern Africa Social Science Research Review*, vol. 18, no. 1, pp. 1–20, 2002.

[16] A. S. Taffesse, P. Dorosh, and S. A. Gemessa, "Crop production in Ethiopia: regional patterns and trends," *Food and agriculture in Ethiopia: Progress and policy challenges*, vol. 93, pp. 53–83, 2012.

[17] G. Ayalew and Y. G. Selassie, "Land suitability evaluation for cereal and pulse crops using geographical information system in east amhara region, Ethiopia," *Research Journal of Agricultural and Environmental Sciences*, vol. 4, pp. 141–148, 2015.

[18] B. Tegene, "Processes and causes of accelerated soil erosion on cultivated fields of South Welo, Ethiopia," *Eastern Africa Social Science Research Review*, vol. 16, no. 1, p. 1, 2000.

[19] A. Gelaw and A. Uncha, "The status of soil quality and indigenous management practices towards the sustenance of soil resource in gamo highlands, southwestern Ethiopia," A Research Report, Arba Minch University, Arba Minch, Ethiopia, 2011.

[20] P. Dorosh and S. Rashid, *Food and Agriculture in Ethiopia: Progress and Policy Challenges*, University of Pennsylvania Press, Philadelphia, PA, USA, 2013.

[21] A. Uncha, "Indigenous soil management techniques and impact of modern agricultural technologies in gamo highlands, southern Ethiopia," Ph. D Dissertation, Department of Geography, University of South Africa, Pretoria, South Africa, 2014.

[22] A. Alemayehu and W. Bewket, "Local climate variability and crop production in the central highlands of Ethiopia," *Environmental Development*, vol. 19, pp. 36–48, 2016.

[23] K. E. Roger, P. K. Y. Bernard, and K. K. Georges, "Soil physico-chemical properties and land suitability evaluation for maize (Zea mays), beans (Phaseolus vulgaris) and Irish potatoes (Solanum tuberosum) in tephra soils of the western slopes of mount Kupe (Cameroon)," *African Journal of Agricultural Research*, vol. 11, no. 45, pp. 4571–4583, 2016.

[24] T. Erkossa, "Land preparation methods and soil quality of a vertisol area in the central highlands of Ethiopia," Doctoral dissertation, University of Hohenheim, Stuttgart, Germany, 2005.

[25] Y. Briza, F. Dileonardo, and A. Spisni, "Land evaluation in the province of ben slimane, Morocco," in *Proceedings of the 21st Course Professional Master: Remote Sensing and Natural Resource Evaluation*, Taipei, Taiwan, December, 2001.

[26] K. Teka and M. Haftu, "Land suitability characterization for crop and fruit production in Midlands of Tigray, Ethiopia," *Momona Ethiopian Journal of Science*, vol. 4, no. 1, pp. 64–76, 2012.

[27] H. Hurni, "Assessing sustainable land management (SLM)," *Agriculture, Ecosystems and Environment*, vol. 81, no. 2, pp. 83–92, 2000.

[28] H. Hurni, W. A. Berhe, P. Chadhokar et al., *Soil and Water Conservation in Ethiopia: Guidelines for Development Agents*, University of Bern, with Bern Open Publishing, Bern, Switzerland, 2016.

[29] A. Alemayehu, M. Maru, and W. Bewket, "Spatial rainfall variability of Oromia region, Ethiopia," *Arsi Journal of Science and Innovation*, vol. 7, no. 1, pp. 48–78, 2022.

[30] J. Warner, T. Stehulak, and L. Kasa, "Woreda-level crop production rankings in Ethiopia: a pooled data approach," *Gates Open Res*, vol. 3, 2019.

[31] Csa (Central Statistical Agency), *Population Size by Sex, Area and Density by Region, Zone and Wereda: July 2022: The Federal Democratic Republic of Ethiopia*, Csa (Central Statistical Agency), Addis Ababa, Ethiopia, 2022.

[32] Csa (Central Statistical Agency), "Agricultural sample survey 2013/2014 (2006 E.C.): volume III, report on farm management practices (private peasant holdings, meher season)," *Statistical Bulletin-. The Federal Democratic Republic of Ethiopia*, Csa (Central Statistical Agency), Addis Ababa, Ethiopia, 2014.

[33] M. A. AbdelRahman, A. M. Saleh, and S. M. Arafat, "Assessment of land suitability using a soil-indicator-based approach in a geomatics environment," *Scientific Reports*, vol. 12, no. 1, Article ID 18113, 2022.

[34] B. N. Anusha, K. R. Babu, B. P. Kumar et al., "Integrated studies for land suitability analysis towards sustainable agricultural development in semi-arid regions of AP, India," *Geosystems and Geoenvironment*, vol. 2, no. 2, Article ID 100131, 2023.

[35] G. Debesa, S. L. Gebre, A. Melese, A. Regassa, and S. Teka, "GIS and remote sensing-based physical land suitability analysis for major cereal crops in Dabo Hana district, South-West Ethiopia," *Cogent Food and Agriculture*, vol. 6, no. 1, Article ID 1780100, 2020.

[36] M. Makungwe, L. M. Chabala, M. Van Dijk, B. H. Chishala, and R. M. Lark, "Assessing land suitability for rainfed paddy rice production in Zambia," *Geoderma Regional*, vol. 27, Article ID e00438, 2021.

[37] M. B. Moisa, F. S. Tiye, I. N. Dejene, and D. O. Gemeda, "Land suitability analysis for maize production using geospatial technologies in the Didessa watershed, Ethiopia," *Artificial Intelligence in Agriculture*, vol. 6, pp. 34–46, 2022.

[38] B. Özkan, O. Dengiz, and İ. D. Turan, "Site suitability analysis for potential agricultural land with spatial fuzzy multi-criteria decision analysis in regional scale under semi-arid terrestrial ecosystem," *Scientific Reports*, vol. 10, no. 1, Article ID 22074, 2020.

[39] H. Yohannes and T. Soromessa, "Land suitability assessment for major crops by using GIS-based multi-criteria approach in Andit Tid watershed, Ethiopia," *Cogent Food and Agriculture*, vol. 4, no. 1, Article ID 1470481, 2018.

[40] Nsrc (National Soil Research Center), "Soils of Sinana agricultural research center: soil survey and land evaluation," Survey Report, National Soil Research Center, Ethiopian Institute of Agricultural Research, Addis Ababa, Ethiopia, 2016.

[41] J. R. Landon, *Booker Tropical Soil Manual: A Handbook for Soil Survey and Agricultural Land Evaluation in the Tropics and Subtropics*, Longman, New York, NY, USA, 1991.

[42] Nma (National Meteorological Agency), "Climate and agroclimate resources of Ethiopia," vol. 1, no. 1, NMA Meteorological Research Report Series, Addis Ababa, Ethiopia, 1996.

[43] D. De la Rosa and C. A. Van Diepen, "Qualitative and quantitative land evaluations," *Land use, land cover and soil sciences*, vol. 2, pp. 59–77, 2009.

[44] S. Moshago, A. Regassa, and T. Yitbarek, "Characterization and classification of soils and land suitability evaluation for the production of major crops at anzecha watershed, gurage zone, Ethiopia," *Applied and Environmental Soil Science*, vol. 2022, Article ID 9733102, 22 pages, 2022.

[45] J. S. Glendinning, *Australian Soil Fertility Manual*, CSIRO Publications, Clayton, Australia, 2000.

[46] T. Everest, G. S. Savaşkan, A. Or, and H. Özcan, "Suitable site selection by using full consistency method (FUCOM): a case study for maize cultivation in northwest Turkey," *Environment, Development and Sustainability*, pp. 1–20, 2022.

[47] E. Fekadu and A. Negese, "GIS assisted suitability analysis for wheat and barley crops through AHP approach at Yikalo subwatershed, Ethiopia," *Cogent Food and Agriculture*, vol. 6, no. 1, Article ID 1743623, 2020.

[48] D. B. Debalke, D. A. Mengistu, and T. E. Admas, "Physical land suitability evaluation of rainfed crop production of wheat, barley, and teff in Arsi zone of Ethiopia," *Arabian Journal of Geosciences*, vol. 16, no. 3, p. 177, 2023.

[49] S. Mohammed, K. Alsafadi, H. Ali et al., "Assessment of land suitability potentials for winter wheat cultivation by using a multi criteria decision Support-Geographic information system (MCDS-GIS) approach in Al-Yarmouk Basin (Syria)," *Geocarto International*, vol. 37, no. 6, pp. 1645–1663, 2022.

[50] T. Everest and E. Gür, "A GIS-based land evaluation model for peach cultivation by using AHP: a case study in NW Turkey," *Environmental Monitoring and Assessment*, vol. 194, no. 4, p. 241, 2022.
