# Supplementary material for: Analyzing land suitability for key cereal crops in Mansa Watershed, Southwest Ethiopia
Source: PLoS One. 2025 Aug 22;20(8):e0328533. doi: 10.1371/journal.pone.0328533 (PMC12373178; doi:10.1371/journal.pone.0328533)
Supplement: Attachment — Submitted filename: Response to editor.docx [file pone.0328533.s003.docx]

Response to editor on a manuscript entitled “Unlocking Agricultural Potential: Analyzing Land Suitability for Key Cereal Crops in Mansa Watershed, Southwest Ethiopia” (PONE-D-23-35036).

We would like to thank the editor for his very useful and constructive comments that have helped us to substantially improve the manuscript confirm the figures submission were created with non-copyrighted sources. We believe to have addressed all comments in the reports. With the specific information requested, we already have information in Table 1 and Table 2 of the manuscript. We have the responses are provided below.

Fig 7. Reclassified elevation map - Software: ArcMap 10.3, ERDAS IMAGINE 2015, IDRISI 17.0, and Google Earth-source <https://earthexplorer.usgs>, Ethiopian Mapping Agency (base map).

Fig 11. LULC cover of the study area- Sentinel-2A image of 2021 from <https://earthexplorer.usgs>, Ethiopian Mapping Agency (base map), Software used: ArcMap 10.3, ERDAS IMAGINE 2015, IDRISI 17.0, and Google Earth.

Fig 12. Reclassified LULC map - Sentinel-2A image of 2021 from <https://earthexplorer.usgs>, Ethiopian Mapping Agency (base map), Software: ArcMap 10.3, ERDAS IMAGINE 2015, IDRISI 17.0, and Google Earth.

Fig 13. Teff suitability map - Ethiopian Mapping Agency, Agricultural Transformation Agency (ATA), Ethiopia National Meteorology Agency (NMA), Software used: ArcMap 10.3, ERDAS IMAGINE 2015, IDRISI 17.0, and Google Earth.

Fig 14. Wheat suitability map- Sources are Ethiopian Mapping Agency (Base map), Agricultural Transformation Agency (ATA) to develop soil OM, pH, Depth, Drainage and Texture map of the study area, Ethiopia National Meteorology Agency (NMA) to interpolate temperature and rainfall data. Software used: ArcMap 10.3, ERDAS IMAGINE 2015, IDRISI 17.0, and Google Earth.

Fig 15. Maize suitability map- Ethiopian Mapping Agency (Base map), Agricultural

Transformation Agency (ATA) to develop soil OM, pH, Depth, Drainage and Texture map of the study area, Ethiopia National Meteorology Agency (NMA) to interpolate temperature and rainfall data. Software used: ArcMap 10.3, ERDAS IMAGINE 2015, IDRISI 17.0, and Google Earth

As aforementioned, more informations is available in table 1 and 2 of the manuscript.
