# Supplementary material for: Analyzing land suitability for key cereal crops in Mansa Watershed, Southwest Ethiopia
Source: PLoS One. 2025 Aug 22;20(8):e0328533. doi: 10.1371/journal.pone.0328533 (PMC12373178; doi:10.1371/journal.pone.0328533)
Supplement: Attachment — Submitted filename: Responses to reviwers.docx [file pone.0328533.s005.docx]

**Journal:** PLOS ONE

**Manuscript number:** PONE-D-23-35036R2

**Title:** Analyzing Land Suitability for Key Cereal Crops in Mansa Watershed, Southwest Ethiopia

We tried to answer the worries forwarded by the reviewers and academic editor as follows:

Dear editor and reviewers,

**Response to Reviewers and editors**

We the authors of this manuscript have tried to address the responses to the comments given to the reviewers and editors questions as follows:

**Editor request1:** Please review your reference list to ensure that it is complete and correct. If you have cited papers that have been retracted, please include the rationale for doing so in the manuscript text, or remove these references and replace them with relevant current references. Any changes to the reference list should be mentioned in the rebuttal letter that accompanies your revised manuscript. If you need to cite a retracted article, indicate the article’s retracted status in the References list and also include a citation and full reference for the retraction notice.

**Answer for editor request1:** Some of the references are amended in the manuscript for instance, see in references 3, 10, 14, 16, 17, and 18 are replaced and references 68 and 69 are added and other ones are improved in reference section (please see in attached track manuscript).

**Reviewer #1** Comment and question:**:**

- Author must reduce abstract size. It must be crispy and to the point. Material and methods must be rewritten (at least one page, discuss in detail)
- Conclusion are suggested to improve

**Answer:** The abstract has reduced in size and methods and conclusions have rewritten and improved (see attached in track manuscript).

**Reviewer #3 comment and question 1:** The methodology is robust and clearly described, but a brief section explaining why factors like market access or irrigation were not included could make the study more comprehensive. This would help expand the discussion on how future work could integrate such socioeconomic factors, which are vital for agricultural planning.

**Answer for reviewer #3; question 1:**

Physical land suitability focuses on the natural characteristics of the land itself, factors like market accessibility and irrigation facilities are part of a broader assessment of land suitability that incorporates human influences and infrastructural considerations. Each type of assessment serves a different purpose in land-use planning and decision-making. Factors like market access or irrigation and other socioeconomic factors which help on future work are integrated and have tried to be discussed in the manuscript which is vital for agricultural planning (see track manuscript in lines 576-578).

**Reviewer #3 comment and question 2:**

The use of AHP for weighting criteria is appropriate, but the subjectivity in weight assignments could be further addressed. Including a small discussion on how expert opinions were selected and any steps taken to reduce bias would strengthen the methodological transparency and provide confidence in the results.

**Answer for reviewer #3: question 2:**

Structured decision-making framework, quantifiable results, Expert-driven insights, consistency checking and	integration with geographic information systems (GIS) were the necessary steps in reducing of subjectivity bias of AHP. AHP is effective for weighting criteria, enhancing the transparency of the expert selection and weight assignment processes can greatly mitigate the subjectivity inherent in decision-making. Employing systematic strategies for selecting experts, reducing bias through consistency checks and sensitivity analyses, and clearly communicating these steps can strengthen the overall reliability of our results derived through AHP. In addition to these AHP provides a more structured, quantifiable, and reliable methodology for land suitability analysis. It allows for expert input, consistency checking, and effective integration with GIS, resulting in more informed decision-making that can readily adapt to changing priorities and enhance spatial analyses.

**Reviewer #3 comment and question 3:**

The spatial resolution of some datasets, especially soil and rainfall, may limit the precision of your findings. Including a sentence or two discussing these limitations and justifying why coarser-resolution data were used, or suggesting the potential benefits of using finer-scale datasets in future studies, would enhance the clarity of the analysis.

**Answer for reviewer #3: question 3:**

The spatial resolution of datasets, particularly for soil and rainfall, can significantly impact the precision of our findings. In this analysis, coarser-resolution data were utilized due to their broader availability and the need for a comprehensive overview at a regional scale. However, the limitations of this approach include potential inaccuracies in localized assessments and variability that finer-scale datasets could capture. Future studies would benefit from integrating higher-resolution datasets, which could provide more precise insights into spatial heterogeneity and improve the reliability of our conclusions. This would allow for a more nuanced understanding of the interactions between soil properties and rainfall patterns, ultimately enhancing the robustness of the research outcomes

Here we attached the revised manuscript with track manuscript changes, clean manuscript, tables in the main manuscript, and all dataset and figure images in separate files.

Best regards

Wakshum Shiferaw (PhD)

Arba Minch University

Phone: +251-911972481
