# Supplementary material for: Analyzing land suitability for key cereal crops in Mansa Watershed, Southwest Ethiopia
Source: PLoS One. 2025 Aug 22;20(8):e0328533. doi: 10.1371/journal.pone.0328533 (PMC12373178; doi:10.1371/journal.pone.0328533)
Supplement: Attachment — Submitted filename: Response to Reviewers.docx [file pone.0328533.s007.docx]

Response to Reviewers

Subject: Sending revised PONE-D-23-35036R4

Title: Analyzing Land Suitability for Key Cereal Crops in Mansa Watershed, Southwest Ethiopia

Journal: PLOS ONE

Editor Comments:

Questions: I don't see the land suitability maps.

Dear Prof. Nguyen-Thanh Son Academic Editor,

Response: We tried to forward the suitability maps with the manuscript and in separate files. W added also the last figure 14 if the manuscript makes clear even though the license issue of the figure requested by an editor of the journal.

Questions: Please make sure that they are presented in the revised manuscript and also discuss about spatial distributions of land suitability classes.

Response: The authors tried revise and discuss spatial distributions of land suitability classes, for instance, look at lines 305-306, 325-327, 342-345, 365-368, 383-386, 403-405, 428-431, 443-445, 470-471, 504-506.

Questions: Please improve the quality and captions of all figures.

Response: Thank you we tried to improve the quality of the figures and figure captions. See in the track manuscript.
